# Three Novel Bacteria Associated with Two Centric Diatom Species from the Mediterranean Sea, *Thalassiosira rotula* and *Skeletonema marinoi*

**DOI:** 10.3390/ijms222413199

**Published:** 2021-12-07

**Authors:** Federica Di Costanzo, Valeria Di Dato, Leonardo Joaquim van Zyl, Adele Cutignano, Francesco Esposito, Marla Trindade, Giovanna Romano

**Affiliations:** 1 Stazione Zoologica Anton Dohrn Napoli, Ecosustainable Marine Biotechnology Department, Villa Comunale, 80121 Napoli, Italy; federica.dicostanzo@szn.it (F.D.C.); acutignano@icb.cnr.it (A.C.); francesco.esposito@szn.it (F.E.); giovanna.romano@szn.it (G.R.); 2 Department of Biology, University of Naples Federico II, Complesso Universitario di Monte Sant’Angelo, Via Cinthia 21, 80126 Napoli, Italy; 3 Institute for Microbial Biotechnology and Metagenomics, Department of Biotechnology, the University of the Western Cape, Bellville, Cape Town 7535, South Africa; ituffin@uwc.ac.za; 4 Istituto di Chimica Biomolecolare (ICB), Consiglio Nazionale delle Ricerche (CNR), Via Campi Flegrei 34, 80078 Pozzuoli, Italy

**Keywords:** diatoms, marine bacteria, metagenome, bioinformatics, secondary metabolites, gene clusters, UPLC-MS/MS

## Abstract

Diatoms are a successful group of microalgae at the base of the marine food web. For hundreds of millions of years, they have shared common habitats with bacteria, which favored the onset of interactions at different levels, potentially driving the synthesis of biologically active molecules. To unveil their presence, we sequenced the genomes of bacteria associated with the centric diatom *Thalassiosira rotula* from the Gulf of Naples. Annotation of the metagenome and its analysis allowed the reconstruction of three bacterial genomes that belong to currently undescribed species. Their investigation showed the existence of novel gene clusters coding for new polyketide molecules, antibiotics, antibiotic-resistance genes and an ectoine production pathway. Real-time PCR was used to investigate the association of these bacteria with three different diatom clones and revealed their preference for *T. rotula* FE80 and *Skeletonema marinoi* FE7, but not *S. marinoi* FE60 from the North Adriatic Sea. Additionally, we demonstrate that although all three bacteria could be detected in the culture supernatant (free-living), their number is up to 45 times higher in the cell associated fraction, suggesting a close association between these bacteria and their host. We demonstrate that axenic cultures of *T. rotula* are unable to grow in medium with low salinity (<28 ppt NaCl) whereas xenic cultures can tolerate up to 40 ppt NaCl with concomitant ectoine production, likely by the associated bacteria.

## 1. Introduction

The role of bacteria in the equilibrium of marine ecosystems is increasingly being recognized, based on the growing number of papers demonstrating their role in organic matter decomposition and their strong interaction with other marine organisms, including diatoms [1,2,3,4,5,6]. Since diatoms are at the base of the marine food web, they are fundamental to the health of the planet, playing a crucial role in carbon fixation [7] and contribute to the formation of oil deposits sinking to the bottom of the oceans due to their heavy frustule [8]. They are represented by more than 200,000 different species able to adapt to every marine niche and bloom in both coastal and oceanic areas, or wherever sufficient nutrients can sustain their growth [8,9].

Studies focusing on diatom–bacteria (D–B) interactions have reported communities of different species of bacteria living strictly on diatoms (strictly associated, SA) or in their close surroundings (free living, FL) [3,6,10,11]. The exact composition of these communities at the species level is not yet well defined and appears to be dependent on different factors, which include the algal species and its growth stages as well as the light and temperature conditions under which they are growing [10,12,13,14]. The D–B interaction is predominantly based on the exchange of metabolites and nutrients (e.g., vitamin B12) [11], including the synthesis of species-specific nutrients, and the secretion of defense molecules against other algal or bacterial species [12,15,16]. An interesting example comes from the bacterial species *Phaeobacter inhibens* that can protect diatoms from harmful prokaryotic species by influencing and shaping the bacterial community developing around them [15,17]. In addition, bacteria seem to establish a stable long-term association with diatoms [14], and certain bacterial orders, in particular Rhizobiales and Rhodobacterales, tend to co-occur with *Symbiodiniaceae* dinoflagellates when establishing symbiosis with other organisms [18]. Moreover, the surface properties of the algal cells can be influenced by SA species that can help in the formation of aggregates [5,6]. Indeed, depending on the type of interaction established, bacteria can contribute to algal aggregation at levels depending on their community composition and the type of algal exopolymer released [5,6,10], which can also serve as a mechanism of escape from predators [5,10].

The high abundance of diverse and novel bacterial species that compose those microbial communities, both SA and FL, could harbor enormous metabolic diversity in which there is hidden potential for the discovery of yet unknown metabolites that could be valuable for exploitation. This is in part due to the space and nutrient limitations that create a competitive environment, requiring bacteria to develop an adaptive response and strategies that enable them to survive over others, including the utilization of secondary metabolites that function as repellents against competing species. Identification and characterization of these metabolites should allow for the discovery of new drugs with application in many fields [19,20]. It has been demonstrated in several cases that the bacterial symbiont, rather than the host organism, is responsible for the production of secondary metabolites beneficial to itself as well as to its host [21]. Some of these compounds have also successfully made it to market [22].

*Thalassiosira rotula* is a cosmopolitan coastal bloom-forming diatom species, present in the Gulf of Naples (Italy) often dominating the phytoplankton community due to its large size and elevated concentration [23]. This species has been explored for the presence of secondary metabolites produced under nutrient stress conditions, i.e., silica depletion, revealing its ability to express genes related to the synthesis of prostaglandins, iridoid type alkaloids and polyketides [24]. *T. rotula* has also been shown to produce compounds toxic for the progeny of predators grazing on it [25] as well as antibacterial compounds [26].

While several studies have been conducted to identify the bacterial species associated with *T. rotula* [14,27,28], to our knowledge, none have explored the biotechnological potential of the bacteria associated with this diatom.

Here, we present the genomes of three novel diatom-associated bacteria reconstructed from a metagenome dataset and investigate their association with three diatom strains. The analysis of their secondary metabolite gene clusters revealed the presence of new hybrid non-ribosomal peptide synthase (NRPS)/Type 1 polyketide synthase (T1PKS) biosynthetic pathways, as well as clusters coding for ectoine, terpenes and bacteriocins.

The availability of these genomes and our preliminary analysis of the association between these bacteria and diatoms will help open the way for an improved understanding of the relationship between these organisms as well as provide the basis for a genomics-based exploration of the secondary metabolite production capability of these bacteria.

## 2. Results

### 2.1. Sequencing, Assembly, Binning and Classification of MAGs

Under antibiotic selection, the sequencing of the associated bacteria from *T. rotula* FE80 genomic DNA (gDNA) resulted in a 22 Mb bacterial metagenome dataset consisting of 416 contigs greater than 5 kb (Table 1).

**Figure 1:**
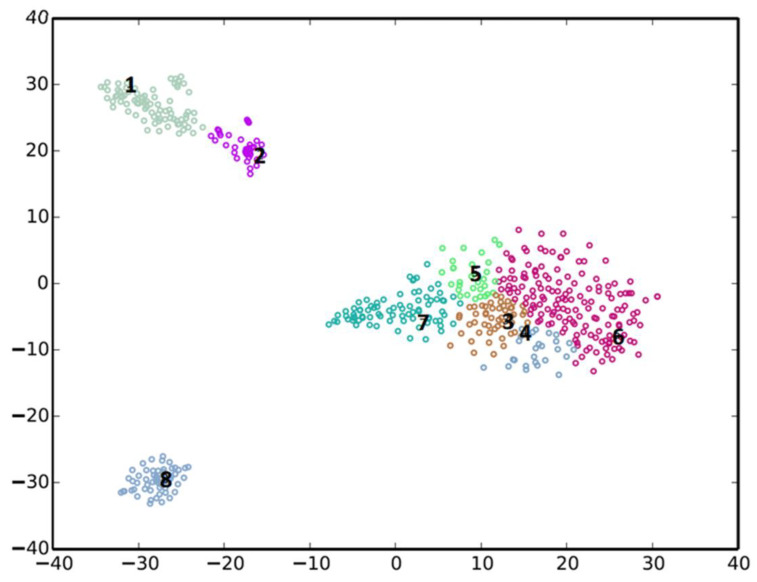
Metagenomic binning of contigs using the MyCC analysis tool [29].

The binning of the metagenomic contigs resulted in eight clusters (Figure 1), three of which (Cl-1, Cl-2 and Cl-8), were well defined and of sufficient completeness and quality to warrant further analysis (Table 2). These were deposited in the Genbank database as high quality draft genomes.

**Figure 2:**
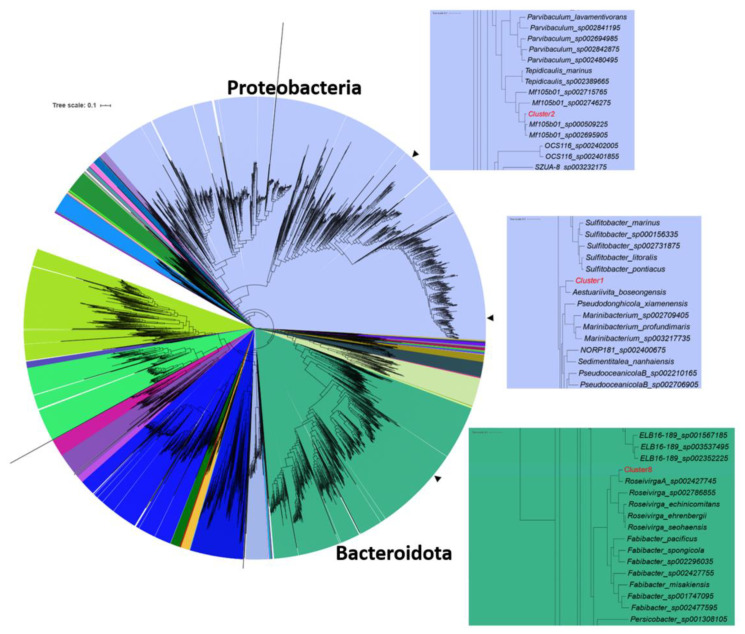
Phylogenetic placement of selected MAGs following analysis performed with GTDB-Tk [33]. The tree is colored by phylum. Arrows indicate the positions of the clusters in the tree and the insets show enlarged views of the region of the tree, including the selected clusters.

With an average amino acid identity (AAI) of 77.56%, 90.05% and 81.41% respectively, taxonomic classification identified Cl-1 as a new species in the genus *Aestuariivita* [30], family *Rhodobacteraceae* (therefore belonging to the larger Roseobacter group); Cl-2 as a new species in the placeholder genus *Mf105b01* [31], family *Parvibaculaceae*; and Cl-8 as a new species in the placeholder genus *Roseivirga_A*, most closely related to the MAG *Roseivirga_A sp002427745*, family *Cyclobacteriaceae* [32] (Figure 2, Table 3, Tables S1–S3).

Based on RAST analysis [34], we identified in Cl-1 the presence of genes encoding a photosystem II-type photosynthetic reaction center and a carotenoid pigment that may interact with the light harvesting system.

Cl-2 was identified as a new species in the genus *Mf105b01* of the *family Parvibaculaceae* that was first described as a bacterial contaminant of the genome sequence of the dinoflagellate *Symbiodinium minutum* [35].

Cl-8 is related to *Roseivirga_A sp002427745* assembled from a coral mucus metagenome [36]. RAST annotation could only place 26% of CDS into a subsystem category for both Cl-8 and *Roseivirga_A sp002427745*, supporting the novelty in the amino acid sequences coded for by these genomes. In addition, RAST analysis showed that Cl-8 encodes 34 putative β-lactamase/cephalosporinases, similarly to *Roseivirga_A sp002427745* that also encodes 30 of these enzymes suggesting that they naturally possess multiple β-lactam resistance mechanisms.

### 2.2. Analysis of MAG Secondary Metabolite Gene Clusters

The potential secondary metabolite production from these organisms was estimated using PRISM [37] and antiSMASH [38] (Table 4 and Table S4). Both pipelines identified similar PKS and NRPS biosynthetic clusters, while antiSMASH provided a broader and more detailed cluster annotation (Table 4 and Table S4).

Cl-2 does not appear to encode any biosynthetic gene clusters, except for one putative bacteriocin, whereas Cl-1 and Cl-8 likely produce new NRPS/PKS hybrid compounds based on the high sequence novelty of the pathways (Figure S1).

### 2.3. Ectoine Quantification in Xenic T. rotula Cultures

Considering the presence of a biosynthetic cluster for the synthesis of the osmolyte ectoine on the Cl-1 genome, we sought to investigate the production and contribution of ectoine by xenic *T. rotula* cultures to osmotic stress tolerance in salinities ranging from 20 ppt to 40 ppt. The ectoine concentration was measured at exponential/early stationary (day 3) and late stationary phases (day 8). Axenic cultures could not tolerate salinities below 28 ppt, while xenic cultures could grow in salinities ranging from 20 ppt to 40 ppt (personal communication).

**Figure 3:**
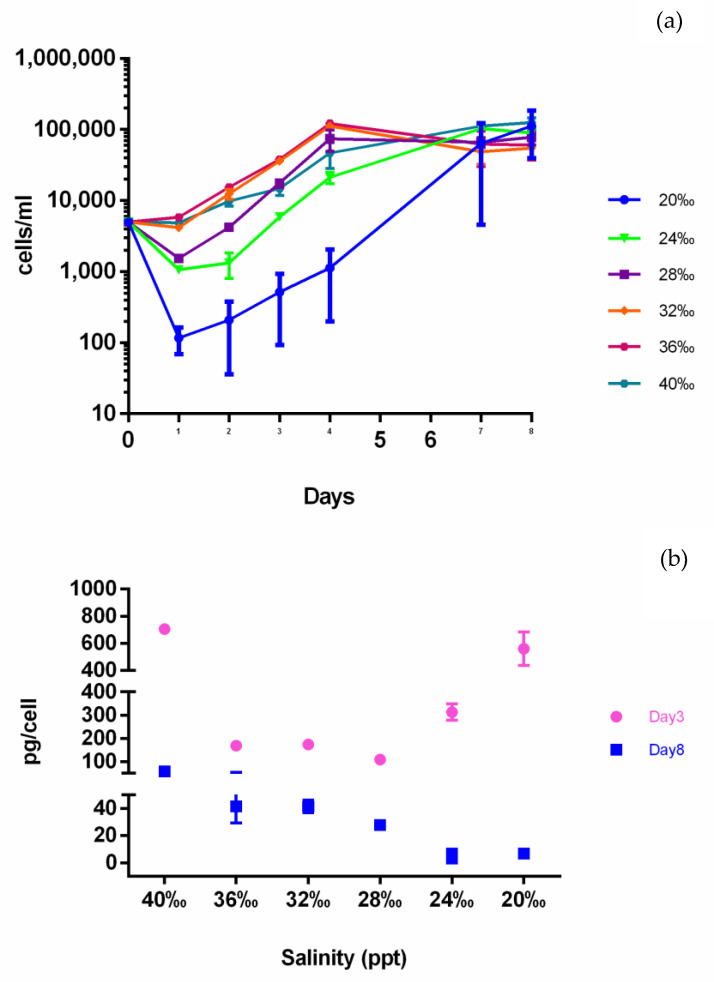
Ectoine quantification in xenic *T. rotula* culture. (**a**) Growth curves of *T. rotula* in media with different salinities; (**b**) ectoine amount, reported as pg per *T. rotula* cell. Day 3 = third day of growth; day 8 = eighth day of growth.

The xenic cultures in higher salinity media (from 32 ppt to 40 ppt) had a similar growth trend, with exponential growth until day 4, while those cultured at 20 ppt, and to a lesser extent those at 24 ppt and 28 ppt, showed a significant reduction in cell numbers during the first day of culturing followed by a recovery period thereafter (Figure 3a). The variability observed for the 20 ppt cultures suggests that the diatoms had difficulty in growing at this low salinity. However, after 8 days, cell density reached similar values at all salinities (Figure 3a).

The ectoine amount was significantly different among different salinities and days, being higher at all salinities at day 3 compared with day 8 (Figure 3b). Interestingly, at day 3, the ectoine expressed in relation to diatom cell number was higher at 20, 24 and 40 ppt, which corresponded to a lower diatom growth rate (Figure 3b). At day 8 of growth in which all the cultures reached the same cell concentrations, ectoine was highest at 40 ppt, stable at 36–32 ppt and decreased at lower salinities (Figure 3b).

**Figure 4:**
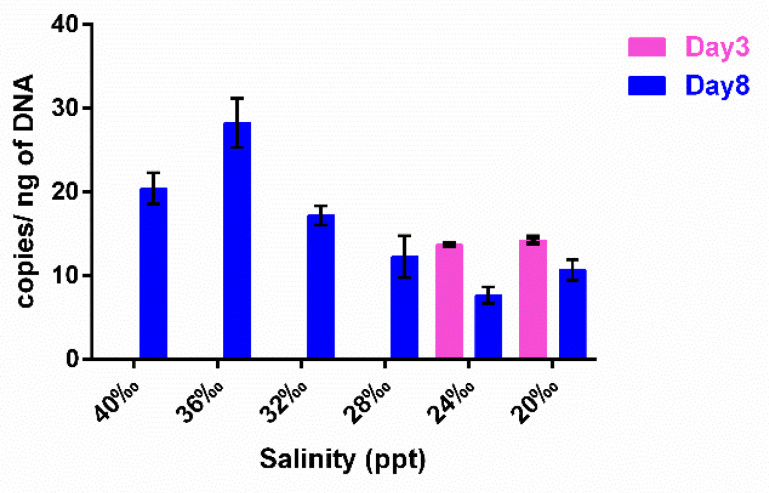
Cl-1 qPCR amplification on SA gDNA extracted from *T. rotula* xenic cultures grown at different salinities. The cultures were sampled at exponential–early stationary phase (Day 3) and at late stationary phase (Day 8) during their growth.

Cl-1 abundance evaluated in these culture fractions by qPCR is shown in Figure 4, where growth was only detected at day 3 and only at the 20 ppt and 24 ppt salinities and at equal amounts (Figure 4, Table S5). On the contrary, at day 8, at all salinities, Cl-1 was clearly detectable (Figure 4, Table S5). The comparison of Figure 3b and Figure 4 shows that, at day 8, the ectoine trend is concomitant with the Cl-1 abundance.

### 2.4. Verification of MAG Association with Diatoms

**Figure 5:**
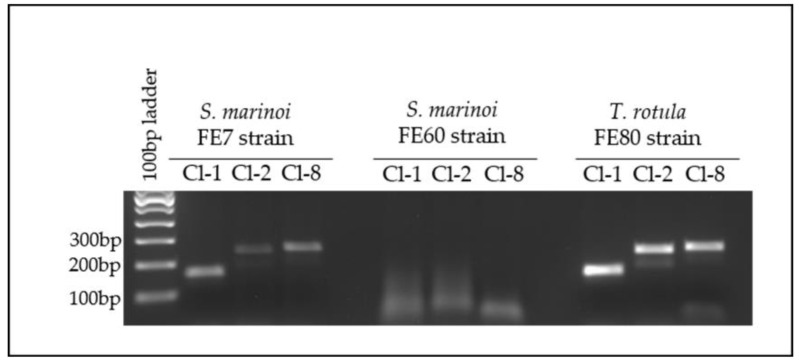
Cl-1, Cl-2 and Cl-8 PCR amplification from FL gDNA. FL fractions of each diatom strain were tested on the 7th day of growth.

Following many unsuccessful attempts to bring the bacteria representing the MAGs into culture, and considering that they could be a co-dependent consortium, we employed PCR and qPCR as a means of tracking their growth and association with the diatom. To confirm the association of the three bacterial strains (Cl-1, Cl-2 and Cl-8) with *T. rotula* FE80, we amplified specific sequences unique to each MAG from gDNA extracted from the associated FL bacterial fraction (Figure 5). In addition, to verify their specific association to different diatom species or their clones, we also amplified from two clones, named FE7 and FE60, of another model centric diatom, *Skeletonema marinoi* (Figure 5). Amplification resulted in specific amplicons from *T. rotula* FE80 and *S. marinoi* FE7 but not FE60, demonstrating a specific clonal preference. Amplification using the Cl-2 specific primer set resulted in two amplicons, both confirmed through sequencing to be specific for Cl-2 (Figure S2b).

**Figure 6:**
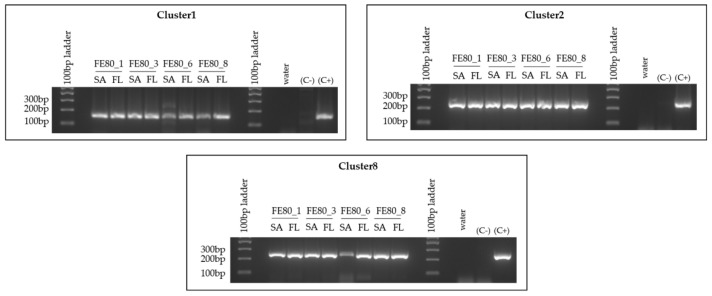
Reintroduction of the bacterial species to *T. rotula* FE80. Cl-1, Cl-2 and Cl-8 PCR amplifications were performed on gDNA extracted separately from the FL and SA fractions associated with the *T. rotula* FE80 cultures after the reintroduction of the *S. marinoi* FE7 associated -FL bacterial fraction. Abbreviations: C+: positive control = *S. marinoi* FE7 xenic total culture gDNA; C-: negative control = *T. rotula* FE80 axenic total culture-gDNA (before reintroduction of bacteria to the culture); FE80_1 = 1 month after the reintroduction; FE80_3 = 3 months after the reintroduction; 6 = 6 months after the reintroduction; FE80_8 = 8 months after the reintroduction of the bacteria. FE80 = *T. rotula* clone FE80.

Since the *T. rotula* FE80 clone we were working with lost viability, the CNR-ICB (Pozzuoli, NA, Italy) laboratories kindly provided us with a different preparation of the same clone. However, their clone was periodically treated with antibiotics that presumably led to the loss of the three MAGs under investigation. We therefore attempted to reintroduce the bacterial assemblage collected from the FL fraction of the *S. marinoi* FE7 cultures to *T. rotula* FE80. The reintroduction appeared successful, and the association was stable as shown by the positive and specific amplification of all three MAG sequences at each time point tested, from 1 month to 8 months after the reintroduction (Figure 6). The absence of diatom species cross-contamination was confirmed through microscopic observation of the culture. Indeed, *S. marinoi* and *T. rotula* are easily differentiated due to the different morphologies and dimension of the two species.

### 2.5. MAG-Diatom Association Type and Temporal Variation

**Figure 7:**
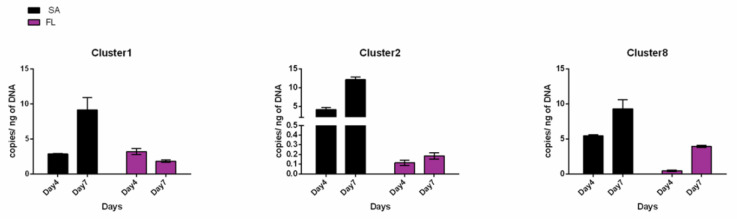
MAG distribution between the SA and FL diatom associated fractions. qPCR using Cl-1, Cl-2 and Cl-8 specific primers performed on SA and FL bacterial fractions collected from *S. marinoi* FE7 sampled on the 4th and 7th days of growth.

qPCR amplification of the MAG sequences from the FL and SA fractions of a *S. marinoi* FE7 xenic culture highlighted a preferential distribution of each MAG in the SA fraction, especially for Cl-2 and Cl-8 (Figure 7). Based on copy number at day 4, Cl-2 and Cl-8 were 11 and 45 times more abundant in the SA than in the FL fraction, respectively (Table S7). No appreciable difference between SA and FL could be detected for Cl-1 on day 4. After 7 days, the abundance of all three MAGs in the SA fraction increased, confirming growth and preferential association with the algae (Figure 7). Conversely, the FL fraction abundance of Cl-1 and Cl-2 did not change with diatom growth. On the contrary Cl-8 abundance also increased in the FL fraction with a copy number ~9 times higher at day 7 compared to day 4 (Figure 7). Among the three MAGs, Cl-2 showed the strongest diatom dependence (SA 67 times higher than FL at day 7) (Figure 7, Table S7).

To further confirm the strict association and assess the possible co-dependence of the MAGs and diatoms, the *S. marinoi* FE7-FL fraction was cultured in three different medium types: marine broth supplemented with *S. marinoi* FE7 spent media (MB), F/2 [39] media supplemented with two different concentrations of sonicated *S. marinoi* FE7 cells (F/2¼ and F/2¾).

**Figure 8:**
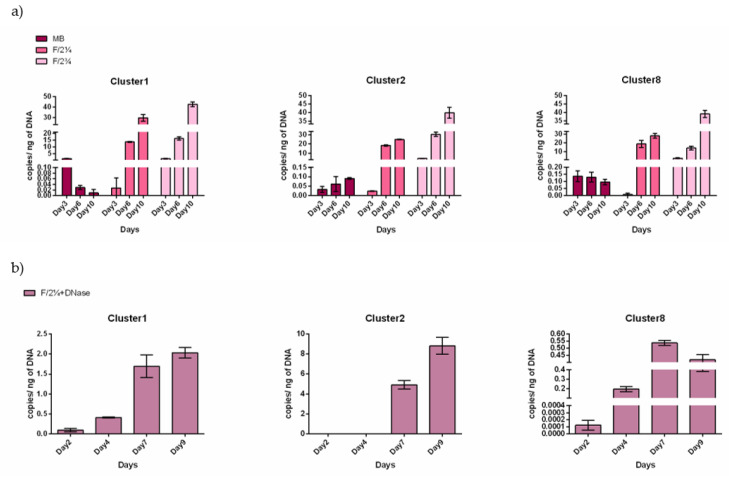
Diatom-free MAG growth. (**a**) qPCR amplification of Cl-1, Cl-2 and Cl-8 specific sequences from gDNA extracted from FL bacteria separated from *S. marinoi* FE7 cultures grown in marine broth supplemented with spent medium (MB), or in F/2 medium supplemented with different concentrations of sonicated *S. marinoi*-FE7 cells (F/2¼ and F/2¾) (see M&M for details). Sampling time points: 3rd (Day3), 6th (Day6) and 10th (Day10). (**b**) qPCR amplification of Cl-1, Cl-2 and Cl-8 on gDNA extracted from FL bacteria grown in F/2¼ media previously treated with DNase. Sampling time points: 2nd (Day2), 4th (Day4), 7th (Day7) and 9th (Day9).

Cultivation in MB medium resulted in a turbid culture, compared to the F/2¼ and F/2¾ cultures when bacterial density was measured spectrophotometrically (Figure S7). However, the relative abundance of the MAGs did not reflect the total bacterial abundance. Cl-1, Cl-2 and Cl-8 detection by gDNA copy number determination revealed their presence when cultured in F/2¼ or F/2¾ medium but not in MB (Figure 8a, Table S9). Indeed, the copy number values from the MB cultures were close to zero at each time point tested, indicating no growth of the three MAGs of interest (Figure 8a). On the contrary, Cl-1, Cl-2 and Cl-8 copy numbers could be detected when the liquid culture was either F/2¼ or F/2¾. The F/2¾ medium was preferred as indicated by the gDNA copy number being very low on day 3 (Figure 8a), indicating that with this media, at least 6 days are needed to detect the growth. This observation was confirmed when quantifying the MAG presence in a *S. marinoi* FE7-derived FL fraction grown in F/2¼ media treated with DNAse (to exclude possible false positives due to gDNA traces in the medium). Indeed, based on gDNA copy number the MAG detection was not due to gDNA traces, but due to actively growing bacteria (Figure 8b, Table S10). The MAG growth curve was thus refined and a different growth rate among the MAGs was highlighted. Cl-1 and Cl-8 had a short growing time, being detectable from day 4 and reaching a maximum at day 7, whereas Cl-2 was detectable from day 7 (Figure 8b). The most abundant was Cl-2 being 4× and 17× more abundant than Cl-1 and Cl-8, respectively.

### 2.6. MAG Antibiotic Tolerance

RAST analysis of the Cl-8 genome revealed 34 putative β-lactamases. Since all three MAGs were recovered from cultures grown in a mixture of antibiotics, including ampicillin, we evaluated their tolerance to two different β-lactam antibiotics (ampicillin (Amp), and cefotaxime (Ctx)) concentrations, when grown in association with algae. In addition, we also tested these antibiotics in combination with the aminoglycoside streptomycin (Str). The tolerance was evaluated on both SA and FL fractions. Two different mixtures of antibiotics were tested: a mixture containing all three antibiotics (ACS mix), and a mix that did not contain Str (AC mix); and both mixtures were tested at two different concentrations (2× and 1×).

**Figure 9:**
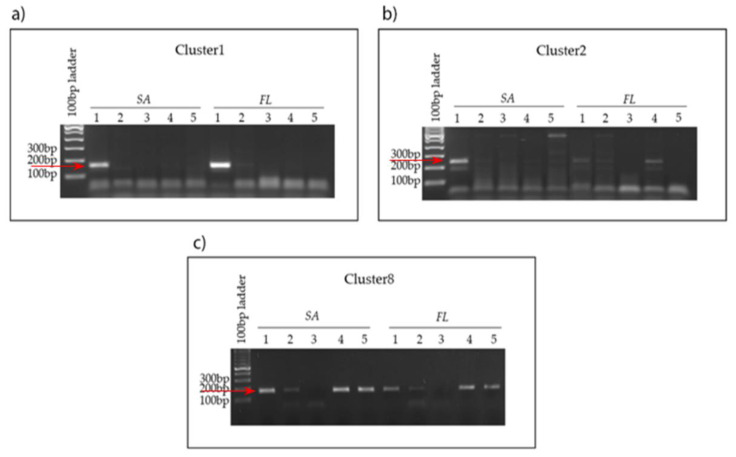
MAG antibiotic tolerance in strictly associated (SA) and free-living (FL) fractions. (**a**) Cl-1. (**b**) C-2 (**c**) Cl-8 detection in the presence of different antibiotic (Abt) mixtures and concentrations. Lanes 1, no Abt; lanes 2, 500 mg/mL Amp, 10 mg/mL Ctx (ACx2); lanes 3, 500 mg/mL Amp, 50 mg/mL Str, 10 mg/mL Ctx (ASCx2); lanes 4, 250 mg/mL Amp, 5 mg/mL Ctx (ACx1); lanes 5, 250 mg/mL Amp, 25 mg/mL Str, 5 mg/mL Ctx (ASCx1). Red arrows indicate expected amplicon size.

Cl-1 was not tolerant to the ACS mix, at either concentration, while it was weakly tolerant to the AC-2× mix (Figure 9a, lanes 2, barely visible band). Similarly, Cl-2 was not tolerant to the ACS mix, weakly tolerant to AC-2× and tolerant to the AC-1×, even if the specific PCR amplification was more visible in the FL than the SA fraction (Figure 9b, SA-FL lane 4).

Cl-8 displayed tolerance to the ACS-1× mix but not to the ACS-2× mix in both fractions (Figure 9c). When Str was not included in the mixture, the antibiotic treatment was tolerated at both concentrations, suggesting that the putative β-lactamases identified in the Cl-8 genome may be responsible for the higher resistance observed for this bacterium (Figure 9c).

## 3. Discussion

Diatoms and bacteria have shared the same environment for more than 200 million years [1] and more than 5% of the diatom genome is composed of bacterial genes [4], whose contribution has been fundamental in the diversification and the evolutionary success of diatoms [1]. Bacteria can live strictly associated to diatom cell walls or in their surrounding water space constituting very different communities [13]. However, in general, the bacterial phyla mainly associated with diatoms are Proteobacteria and Bacteroidetes [40] with few genera representative members, such as *Sulfitobacter*, *Roseobacter* and *Flavobacterium* [1].

Despite the importance of these associations in the evolutionary history of diatoms, diatom–bacteria interactions remain poorly studied [41]. Recently however, this field has garnered more attention, and studies are performed based on the analysis of the entire microbiome associated with diatoms, reporting a global perspective on their relationships [42].

Within this work, based on metagenome analysis of bacteria associated with *T. rotula* we identified three new bacterial species, named Clusters 1, 2 and 8 (Cl-1, Cl-2, Cl-8). The three selected MAGs are members of the families *Rhodobacteraceae*, *Parvibaculaceae* and *Cyclobacteriaceae*, known to be present in the marine environment, which have been shown to have a strong association with *Symbiodiniaceae* [18,35], while their association with the Bacillariophyceae has never been described before. These findings add novelty to what is currently understood about the bacterial species associated with diatoms. Supporting these species’ novelty, for example, Cl-8, defined as a new species closely related to the *Roseivirga* genus, shared only 26% of its CDS with the MAG *Roseivirga_A sp002427745* (81.41% AAI) belonging to the *Cyclobacteriaceae* family [43].

In their 2020 study, Mönnich and coworkers identified *T. rotula* associated bacterial communities to be dominated by *Rhodobacteraceae* (30.5%), *Alteromonadaceae* (27.7%), and *Oceanospirillales* (18.5%) [14]. The species identified in our work would not have been detected in that study, as their methodology could, at best, classify bacteria at family level and was based on an older database (SILVA v128 September 2016). This database did include the 16S rRNA sequence for *A. boseongensis*, but not the closest relatives of Clusters 2 and 8. The coarse grained-approach of identifying the major groups of bacteria associated with any environment using single marker genes has utility for environmental monitoring, but shows its limitations here as little can be said about the interactions between symbionts at this level (family or genus) and shows the advantage of performing whole genome sequencing and employing the latest databases available as opposed to targeting just the 16S rRNA marker.

Based on taxonomic classification Cl-1 was assigned to the *Aestuariivita* genus that currently consists of only two described members, namely *A. boseongensis*, isolated from a Korean tidal flat sediment in 2014 [30] and *Aestuariivita atlantica* isolated from the deep sediment of the Atlantic Ocean in the 2014 [44]. A phylogenomic analysis by Wirth and Whitman in 2018 reassigned *A. atlantica* to the novel genus *Pseudaestuariivita* with the type species *Pseudaestuariivita atlantica*, separating it from *A. boseongensis* [45]. This recent reassignment of genera and species highlights a gap in the classification of these genera which could include a number of novel and as yet unexplored species, such as Cl-1. The presence of genes encoding a photosystem II-type photosynthetic reaction center in Cl-1 suggested a photoheterotrophic behavior for it, although no PufX (component of the reaction center–light-harvesting 1–PufX (RC–LH1–PufX) complex) homolog could be identified. Although essential to enable photosynthesis in some organisms, such as *Rhodobacter sphaeroides*, in others, such as *Thermochromatium tepidum*, it is not required [46,47]. Its closest relative, *A. boseongensis* carries the same complement of photosynthesis reaction center genes, with the exception of the light-harvesting protein B-800/850 alpha and beta subunits. However, enzyme homologs involved in CO_2_ fixation or carboxysome formation were not found, similar to many other aerobic anoxygenic photoheterotrophic bacteria, including the model organism *Dinoroseobacter shibae* which has been shown to use the 3-hydroxypropionate cycle for CO_2_ fixation [48,49]. The key enzymes needed for the 3-hydroxypropionate cycle, (crotonyl-CoA carboxylase and propionyl-CoA carboxylase), together with the rest of the enzymes in the pathway are present in both Cl-1 and *A. boseongensis*.

The prediction for carotenoid pigment production in Cl-1 also supports its hypothesized photoheterotrophic nature and may interact with the light harvesting system. The phytoene synthase and dehydrogenase, responsible for carotenoid production, are divergently transcribed from two of the subunit genes (*chlI* and *chlD*) that compose the magnesium chelatase, essential for installing the Mg^2+^ ion in the bacteriochlorophyll protoporphyrin IX ring. This suggests that the regulation of carotenoid expression is linked to production of the chelatase responsible for bacteriochlorophyll formation and could be oxygen dependent, as observed for *R. sphaeroides* [50]. The *Rhodobacteraceae* are known to have phototrophic members that can be very flexible in their energy generation using chemotrophic paths if needed [50,51].

Evidence of the association of a Cl-2-like organism with *T. pseudonana* exists in the genome GCA_002380265.1 deposited on the Genbank database. Bacteria identified through 16S rRNA sequencing as belonging to the genus *Mf105b01* were found to be associated with non-axenic *T. pseudonana* cultures (strain CCMP1335, National Center for Marine Algae and Microbiota (NCMA)), an isolate collected in 1958 from Moriches Bay (Long Island, New York) and maintained in continuous culture [31]. The genome was originally recovered from the Tara Oceans expedition data, SRA ERR599368, collected at station TARA_111 in the South Pacific Ocean [36,52]. In 2019, members of the *Mf105b01* genus were identified in the bacterial fraction associated with *T. pseudonana* [31], growing both under standard culture conditions and in the presence of oil, representing between 14% and 25% of the bacterial fraction strictly associated to the algae [31]. The placement of the Cl-2 species in the *Mf105b01* genus provides further support to consider that members belonging to this genus could all have close associations with phytoplankton. This is not yet well explored and warrants further investigation.

Physiological characterization of the MAGs evidenced differences in their growth rates, distribution in the diatom culture and antibiotic tolerance. An interesting observation is that the presence of all three bacteria in both *S. marinoi* FE7 and *T. rotula* FE80, and absence in *S. marinoi* FE60, cultures suggests that these may form a cohort that co-occur with a specific host. The microbial consortium showed a clear dependence on diatom organic matter when cultured in a liquid medium. In F/2 medium supplemented with sonicated diatoms, Cl-1 and Cl-8 needed at least four days, while Cl-2 needed at least seven to be detectable by qPCR, whereas little to no growth was observed in supplemented marine broth. This differs to what is present in the literature regarding the genus closest to Cl-1 [30] and Cl-8 [53,54,55,56,57], which were isolated and maintained on marine agar and marine broth, while all our attempts to culture Cl-8 on marine agar medium (including supplementation with diatom organic matter) were unsuccessful. This preference for diatom organic matter and their major distribution in the SA fraction of a classical diatom culture supports their dependence on molecules produced by the diatoms or the existence of a mutualistic relationship. Relationships based on nutrient exchange are extensively documented in literature. Diatoms release or passively exudate amino acids, carbohydrates and organic acids that are attractants for bacteria [58], while bacteria produce and release vitamins, ammonium or organosulfur molecules in exchange for organic matter released by diatoms [2,11,59].

Another difference among the three selected MAGs comes from the tolerance to β-lactam and aminoglycoside antibiotics. Cl-1 and Cl-2 were weakly tolerant to ampicillin and cefotaxime (β-lactam type antibiotics) and sensitive to streptomycin, whilst Cl-8 was tolerant to both β-lactam type antibiotics and streptomycin (Str) in a dose-dependent manner. These results provide functional validation of the in silico prediction by RAST (β-lactamase) which identified the presence of 34 putative genes for β-lactamase/cephalosporinases in the Cl-8 genome. This could be a common feature of the *Cyclobacteriaceae* [32], excluding the possible acquisition through horizontal transfer during culturing. However, with such a significant β-lactamase capacity, it could be considered surprising that C1-8 was not tolerant under all antibiotic mixtures tested. β-lactamases hydrolyze the β-lactam ring giving resistance to β-lactam antibiotics. However, Str can inhibit the β-lactamase enzymes, overcoming bacterial resistance [60]. The interplay between these antibiotics on the bacterial resistant machineries could explain the dose-dependent tolerance of Cl-8 to the antibiotic mix containing Str.

Functional annotation by PRISM [37] and antiSMASH [38] of pathways coding for secondary metabolites revealed the presence of biosynthetic clusters belonging to polyketides (PKS), non-ribosomal peptides (NRPs), terpenes (TP) and antibiotic classes of molecules. Moreover, the uncharacterized (novel) hybrid NRPS/PKS pathways annotated in the Cl-8 genome make it interesting for the search of molecules useful in pharmacological applications.

Among the other functional annotations, we focused our attention on the ectoine biosynthetic cluster identified in Cl-1. Ectoine is one of the compatible solutes able to accumulate in cells in response to external increases in osmotic pressure without compromising cell physiology. Other than serving as osmotic balancing agents, compatible solutes also show direct protective effects against freezing, high UV irradiation, high temperature, and macromolecule denaturation under adverse conditions [61,62,63,64]. Not all of them are present in all organisms; in fact, their occurrence seems be spread out and sometimes species specific, with some functional specialization. As an example, neutral zwitterionic compatible solutes such as, among others, ectoine are frequent in many mesophilic bacteria, whilst negatively charged organic solutes such as di-myo-inositolphosphate and mannosylglycerate (MG) have often been identified in hyper/thermophilic bacteria and archaea [65,66,67,68]. Based on these observations, we decided to verify the functionality of the ectoine biosynthetic cluster by measuring the ectoine produced in xenic *T. rotula* cultures under salinity stress. Our results indicated that ectoine is produced in the culture and increases under both hypo- and hyperosmotic shock. Interestingly, while axenic *T. rotula* cultures did not survive at salinities below 28 ppt, xenic cultures were able to grow at all the salinities tested and those exposed to 20 ppt salinity were able to overcome the stressful conditions and recovered their growth. The fact that axenic cultures were able to grow at 40 ppt salinity indicates that other mechanisms inside the diatom can compensate for high salinity stress.

In xenic cultures, there is increased ectoine levels at both very low and high salinities (20 ppt and 40 ppt). The capacity of Cl-1 to produce ectoine could resemble the ectoine production in *Halomonas elongata* which, when exposed to both low and high salinities, produces increased quantities of compatible solutes [69]. Thus, C1-1 could be complementing ectoine production under both hypo- and hyperosmotic conditions (20 ppt and 40 ppt). This phenomenon is actually used for the commercial production of ectoine [69] in a process called “bacterial milking”. Looking at the ectoine levels reported in Figure 3, it looks like when fewer diatom cells are in the culture, more ectoine is produced. It would be of interest to determine if the activation of ectoine production in Cl-1 is regulated independently of the diatom host or through signaling from the diatom. Ectoine production in diatom culture was reported recently by Fenizia and coworkers who reported ectoine in *Thalassosira weissflogii* xenic cultures as being produced by the associated bacteria with a very small contribution by the algae [70]. In addition, Vallet and coworkers used the presence of ectoine and absence of choline as metabolic markers for the presence of bacteria in association with algae (*Ulva*) [71].

Cl-1 abundance in the *T. rotula* xenic cultures in the SA fraction on the 8th day of growth correlated with ectoine production. The highest copy number corresponded with higher ectoine concentrations (36 ppt and 40 ppt), decreasing with decreasing salinity, similarly to ectoine levels. On the other hand, at the 3rd day of growth, Cl-1 was undetectable, except at 20 ppt and 24 ppt salinities, which coincides with the higher ectoine concentrations measured. As another ectoine biosynthetic cluster was found in the total metagenome (Table S12), we hypothesize that other bacterial species can contribute to the ectoine production in the culture that also supports microalgae growth at 40 ppt salinity. Alternatively, since Cl-1 was undetectable early on in the growth cycle, it could suggest that, if it is responsible for most of the ectoine production, the few cells that are present are expending most of their resources to produce ectoine.

## 4. Conclusions

With our work, we identified three new bacterial species associated to at least two diatom species, i.e., *Skeletonema marinoi* and *Thalassiosira rotula*. These bacteria showed diatom clonal preferences and the need for a strict association with them. They appear to contribute to the diatom adaptive response with the supply of osmolite and antibiotic resistance and have shown potential for the production of biotechnologically interesting secondary metabolites.

This work serves as a preliminary characterization of these bacteria that opens the door to further exploration of the diatom–bacteria relationship and to the biotechnological development of new and possibly pharmaceutically relevant molecules.

## 5. Materials and Methods

### 5.1. Strain Information and Culturing

*Thalassiosira rotula* strain FE80 was isolated in 2011 in the Gulf of Naples (40°48.5′ N, 14°15′ E), Mediterranean Sea (Italy) and the *Skeletonema marinoi* strain FE7 was isolated from phytoplankton samples collected during diatom blooms in the northern Adriatic Sea in 1997 [72]. Clonal cultures were established by isolating single cells or short chains from phytoplankton net samples collected from the surface layer of the water column. Cultures were grown in sterile filtered oligotrophic seawater at 36 ppt amended with F/2 [39] nutrients and maintained at a temperature of 20 °C, at 12:12 h light:dark cycle, and with a photon flux of 100 μmol photons m^2^ s^−^^1^.

To remove excess bacteria from *T. rotula* FE80 diatom cultures, 250 mL of exponentially growing culture was inoculated in F/2 medium containing final concentrations of 2 mg/L streptomycin (PanReac AppliChem, A1852, Deltek srl Pozzuoli (Na), Italy), 2 mg/L penicillin (PanReac AppliChem, A1837, Deltek srl Pozzuoli (Na), Italy), 10 mg/L ampicillin (PanReac AppliChem, A0839, Deltek srl Pozzuoli (Na), Italy) and allowed to grow for six days under standard growth conditions. After six days, the culture was supplied with fresh F/2 media, containing antibiotics, to a final volume of 1 L and cultivation continued for seven more days. Cells were collected on a 1.2 µm RAWP membrane filter (Millipore, Billerica, MA, USA). The filter was rinsed with 1.5 mL seawater to detach the cells that were further collected into Eppendorf tubes by centrifugation at 3800× *g* at 4 °C for 5 min (Eppendorf Centrifuge 5810 R). The pellet free of supernatant was immediately frozen in liquid nitrogen and stored at −80 °C.

For ectoine and Cl-1 quantification, 5000 cells/mL were inoculated in 2 L of F/2 medium at different salinities, each in triplicate. These media were made as follow: 2 L of F/2 medium prepared with sterile seawater at 38 ppt was supplemented with 4 g of NaCl to obtain a final salinity of 40 ppt. Starting from this medium, the other salinity points were obtained by dilution with autoclaved Milli-Q water. All the salinities were checked before and after sterilization using a portable refractometer. After three days, 500 mL of each culture were collected by filtration on 3 µm polycarbonate filters (Millipore, Billerica, MA, USA). After eight days of growth, cells from 500 mL of culture were collected by centrifugation at 1900× *g* for 10 min at 4 °C. All the samples collected at the two time points were immediately frozen in liquid nitrogen and stored at −80 °C until gDNA extraction (see “DNA extraction, primer design and PCR” paragraph for details) or ectoine quantification (see “Ectoine quantification by UPLC-MS/MS” paragraph for details).

### 5.2. Ectoine Quantification by UPLC-MS/MS

Ectoine was measured applying a newly developed quantitative UPLC-mass spectrometry-based methodology by using an internal standard calibration approach.

LC-MS analyses were performed on an Acquity UPLC System (Waters, Milford, MA, USA) coupled to a 3200 API Triple Quadrupole mass spectrometer (Sciex, Foster City, CA, USA) with a Turbo VTM interface equipped with a turbo ion spray probe used in positive ion mode. The chromatographic analysis was developed on a Luna Omega Polar (100 × 2.1 mm, i.d. 1.6 µm, Phenomenex, Bologna, Italy) by using as eluent A water 0.1% FA and eluent B ACN 0.1% FA; a gradient elution was applied from A 100% to 70% in 3 min, returning back at 100% A in 0.1 min. A re-equilibration step of 1 min was included before successive runs. The flow rate was set at 0.45 mL/min. Separations were performed at a temperature of 35 °C, the autosampler was maintained at 10 °C and injections were of 1 µL. A multiple reaction monitoring (MRM) experiment was used to collect data, by setting the following source parameters: curtain gas (N_2_), 20 psi; ion source gas (GS1), 55 psi; turbogas (GS2), 70 psi; desolvation temperature, 550 °C; collision activated dissociation gas (CAD), 4 a.u.; and ion spray voltage, 5500 V. For ectoine, the following transitions were monitored: 143 > 68 (quantifier) and 143 > 97 *m*/*z* (qualifier). A calibration curve was constructed in the range 2.5–250 ng/mL. Phenylalanine-d5 (Phe-d5) was used as internal standard (IS, 1 µg/mL) and monitored at 171 > 125 *m*/*z*. Analyst software (version 1.6.2; SCIEX) was used for data acquisition. Multiquant software (version 2.1.1, SCIEX) was used for quantitative analysis.

Cell pellets or filters from 500 mL cultures, in triplicate for each experimental condition (see “Strain and cell cultures” paragraph for details), including a control batch, were extracted with MeOH (3 × 5 mL). Phe-d5 was added as IS to obtain a concentration of 1 µg/mL in the final sample. Methanolic extracts were reconstituted in 90:10 (water/MeOH) before LC-MS analysis.

### 5.3. DNA Extraction and Sequencing

The collected *T. rotula* FE80 was used to extract mixed genomic DNA (mgDNA) from both algae and the associated bacteria according to the DNeasy Plant Maxi Kit (Qiagen, Cat. No. 68163, S.I.A.L. Srl, Rome, Italy) handbook. One nanogram of total genomic DNA was used to prepare one NexteraXT library (FC-131-1024; Illumina, Hayward, San Diego, CA, USA). The resultant library was sequenced on the Illumina MiSeq platform using a MiSeq Reagent kit V2 (MS-102-2003; 500 cycle) to generate paired end reads (2 × 250 bp), including a 10% PhiX Control v3 (FC-110-3001) as per the manufacturer’s recommendation.

### 5.4. Metagenome Sequencing, Assembly, Binning and Annotation

Reads were processed by first mapping to the phi-X174 genome and removing reads that were not correctly demultiplexed. Only reads in pairs were taken further for assembly and singletons discarded. Next, reads containing any ambiguous nucleotides were removed. The remaining read pairs were merged. The merged and un-merged pairs, as well as singletons from the ambiguous nucleotide trim were co-assembled.

Assembly was performed using CLC Genomics Workbench version v7.5.1 (Qiagen, http://www.clcbio.com) with the length fraction set at 0.8 and similarity fraction at 0.9. Mismatch insertion and deletion costs were left at their default values. The “global alignment” and “update contigs” settings were enabled, while scaffolding was turned off. The word and bubble sizes were set to automatic mode as well as paired distance detection.

The binning of metagenomic contigs was performed using MyCC (https://tinyurl.com/w8e4ge6, version 2017) [29] with 5 mer and meta settings, while completeness and contamination of metagenome-assembled genomes (MAGs) as well as genome quality were determined using CheckM using the lineage-specific workflow and default parameters [73].

Annotation of MAGs was performed using Prokka v1.1.2 [74] through the KBase [75] online analysis platform as well as Rapid Annotations using Subsystems Technology (RAST; https://rast.nmpdr.org/, access date 18 March 2020). The quality of the assembled MAGs was determined using CheckM as required by the MIMAG (Minimum Information about a Metagenome-Assembled Genome) guidelines [76].

Whole genome phylogeny was determined using the Genome Taxonomy Database tool kit (GTDB-Tk) release 89 implemented in KBase [48] and the output maximum likelihood tree visualized using iTOL (https://itol.embl.de/). tRNAs were detected using the ARAGORN webserver [77]. MAGs were analyzed for the presence of biosynthetic gene clusters using PRISM [37] and antiSMASH v5.1.1 [38].

### 5.5. SA and FL Bacterial Fractions Separation

To obtain the two bacterial fractions, xenic *S. marinoi* FE7 cultures were subjected to two subsequent centrifugation steps: 1900× *g* for 10 min at 4 °C to collect the strictly associated fraction (SA) and 10,200× *g* for 30 min at 4 °C in falcon followed by 8950× *g* for 30 min at 4 °C in Eppendorf tubes (Beckman Avanti^TM^ 30 Centrifuge, Beckman Coulter srl, Milano, Italy) to collect the free living (FL) bacteria. The collected samples were immediately frozen in liquid nitrogen and stored at −80 °C until use.

### 5.6. Reintroduction of Bacteria in T. rotula FE80 Cultures

The axenic *T. rotula* FE80 culture was provided by CNR-ICB (Pozzuoli, NA, Italy) laboratory. A total of 100 μL of FL bacteria prepared from a xenic *S. marinoi* FE7 culture and stored in a cryovial with 40% glycerol were inoculated in a 30 mL axenic *T. rotula* FE80 culture at exponential growth phase. The culture was maintained at a temperature of 20 °C, at 12:12 h light:dark cycle, and with a photon flux of 100 μmol photons m^2^ s^−^^1^ for 3 days, which was then refreshed by a 1:30 dilution with fresh F/2 medium. When stabilized, the culture was refreshed weekly in the same manner and the presence of the bacteria was periodically checked with PCR on the SA-FL fraction gDNA preparations (see “DNA extraction, primer design and PCR” paragraph for details).

### 5.7. Cl-1, Cl-2 and Cl-8 Growth Characterization

Xenic cultures were harvested after 0, 4, and 7 days of growth and bacterial fractions were separated as described before.

For the antibiotic tolerance test, exponentially growing xenic *S. marinoi* FE7 cultures were diluted 1:10, in a final volume of 100 mL, with F/2 medium containing different combinations of the following antibiotics, ampicillin (Amp) (PanReac AppliChem, A0839), cefotaxime (Ctx) (Sigma Aldrich C7039) and streptomycin (Str) (PanReac AppliChem, A1852), and allowed to grow for 72 h. At the end of the 72 h, cultures were again diluted 1:10 into F/2 medium, antibiotic treated as above and allowed to grow for 7 days. Two antibiotic combinations and concentrations were used:

ACx2: 500 mg/mL Amp; 10 mg/mL Ctx;

ACSx2: 500 mg/mL Amp; 10 mg/mL Ctx; 50 mg/mL Str;

ACx1: 250 mg/mL of Amp; 5 mg/mL Ctx;

ACSx1: 250 mg/mL of Amp; 5 mg/mL Ctx; 25 mg/mL Str.

At the end of the treatment, cultures were harvested as described before.

### 5.8. Liquid Bacterial Cultures

FL bacteria (collected as described in the paragraph “SA and FL bacterial fractions separation”) were diluted 1:10 in a final volume of 150 mL of two different media: F/2 medium supplemented with sonicated diatoms or Marine Broth (2216 DIFCO) supplemented with a 1:1 ratio of spent medium. The spent medium was collected from a *S. marinoi* FE7 culture at the 7th day of growth and then was filtered onto 0.22 μm pore size filters (SLGS033SB Millipore). A total of 150 mL of F/2 medium supplemented with sonicated diatoms was obtained as follow. After 7 days of growth, 150 mL of cultures were harvested by centrifugation at 1900× *g*, 10 min, 4 °C. The resulting supernatant was used to collect the FL bacterial fraction. The pellet was resuspended in 2 mL of F/2 and sonicated on ice for 3 pulses of 30 s at 40 hrz, then 30 s on ice. Then 0.5 mL of the sonicated material was resuspended in 149.5 mL of F/2, resulting in a ¼ of the final diatom cell culture concentration. The remaining sonicate material, 1.5 mL, was also resuspended in 148.5 mL of F/2, obtaining a second concentration corresponding to ¾ of the final diatom cell culture concentration. The ¼ sonicate concentration was also used for cultures in which 100 μL of the undiluted FL bacteria was inoculated into 8 mL of F/2 supplemented with sonicate treated with DNAse (1 μL for each 30 μL of sonicate) and incubated at 37 °C for 30 min before addition to the F/2 medium. All the cultures were maintained at a temperature of 20 °C, at 12:12 h light:dark cycle, and with a photon flux of 100 μmol photons m^2^ s^−^^1^.

### 5.9. Solid Media Culturing

FL bacteria, isolated as described before, were serially 1:10 diluted in F/2 medium and spread on different types of solid media: Marine Broth with 1.4% agar (European Bacteriological Agar, Condalab) and a second medium prepared with F/2 containing 1.4% agar and once solidified was subsequently covered with sonicated diatoms (¼ concentration) treated with DNAse as described above. These media were also prepared by replacing the agar with 0.8% Gellan-Gum (Gelrite, SERVA) as the gelling agent. Gellan-Gum was dissolved in Milliq water to a 2× concentration (1.6 gr in 100 mL corresponding to 0.8%) and autoclaved. Soon after, one volume of Gellan-Gum was mixed with one volume of Marine Broth or F/2 (2×, previously autoclaved) under stirring at 80 °C, and poured into petri dishes (25 mL for each plate). FL bacteria were spread on top with a metallic T-shaped spreader or streaked with a wire loop and allowed to grow at temperatures of 18 °C and 28 °C, with a 12:12 h light:dark cycle and in complete darkness, from 48 h to 10 days. Then, 10 to 50 colonies from Marine Broth plates were screened by PCR to search for Cl-8 positive colonies (see “DNA extraction, primer design and PCR” paragraph). Colonies on algae-supplemented-F/2 (sonicated diatoms) plates were collected by scraping since they never reached a size to be easily collected. The material collected in this way was treated as a colony for the following PCR analysis.

### 5.10. DNA Extraction, Primer Design and PCR

Genomic DNA (gDNA) was extracted by vortexing diatom/bacterial pellets with 0.4 g of 0.2–0.3 mm glass beads (G1277-Sigma-Aldrich), 0.5 mL of phenol (PanReac AppliChem, A1153,0100) and 0.5 mL of TE buffer (10 mM Tris HCl pH 7.6 and 1 mM EDTA pH 8.0). DNA extraction was performed as reported in [78]. SA fraction gDNA was extracted from xenic culture pellets of mixed diatom and bacterial cells. FL fraction gDNA was extracted from the pellet deriving from centrifugation of the xenic culture supernatant. Primer3 software V. 0.4.0 [79,80] was used to design PCR primers specific for sequences on the Cl-1, Cl-2 and Cl-8 contigs (see table below), while 16S rRNA universal oligo primers (E9F/U1510R) were taken from [81,82]. Oligo sequences (see Table 5) are listed below:

Each sequence was tested by PCR in a 25 μL final reaction volume with 2.5 μL of 10× PCR reaction buffer (Roche, Basel, Switzerland), 2.5 μL of 2 mM dNTP, 0.3 μL of 5 U/μL Taq (Roche, Basel, Switzerland), 0.25 μL of 1% DMSO, 1 μL 10 μΜ of each oligo, 1 μL of 50 ng/μL DNA template and nuclease-free water up to 25 μL. The PCR was conducted using the T100 Thermal cycler (Bio-Rad Laboratories, Hercules, CA, USA) and the PCR program consisted of a denaturation step at 95 °C for 3 min, 35 cycles at 95 °C for 30 s, 55 °C 30 s, 72 °C for 30 s, and a final extension step at 72 °C for 7 min. For PCR on colonies, the template was obtained as follows: each colony or scraped colony was resuspended in 20 μL of TE buffer (10 mM TrisCl pH 7.6 and 1 mM EDTA pH 8.0), heated at 95 °C for 3 min and centrifuged at 12.000 rpm for 2 min (Eppendorf Mini Spin Centrifuge). Then, 1 μL of the supernatant obtained was tested by PCR using the same temperature cycle and reaction mix described above. Amplified PCR products were analyzed by 1.5% agarose gel electrophoresis and the resulting bands were excised from the gel and extracted according to the GenElute Gel Extraction Kit protocol (Sigma-Aldrich, St. Louis, MO, USA). See Supplementary Materials for uncropped gel images in Figures S2a, S3, S6 and S8.

Sequences were obtained by BigDye Terminator Cycle Sequencing Technology (Applied Biosystems, Foster City, CA, USA) and purified using the Agencourt CleanSEQ Dye terminator removal Kit (Agencourt Bioscience Corporation, Beverly, MA, USA) in an automated robotic station Biomek FX (Beckman Coulter, Pasadena, CA, USA). Products were analyzed on the Automated Capillary Electrophoresis Sequencer 3730 DNA Analyzer (Applied Biosystems, Foster City, CA, USA). Sequences were analyzed with Chromas v 2.6.6 (http://technelysium.com.au/wp/chromas, accessed date: April to June 2021). Identity of the retrieved sequences was verified with the standalone setup BLAST+ v 2.11.0 (https://ftp.ncbi.nlm.nih.gov/blast/executables/LATEST/, accessed date: April to June 2021). See Supplementary Materials for the sequences of the cluster-specific genes and 16S rRNA gene.

### 5.11. Quantitative PCR Analysis

Quantitative PCR (qPCR) experiments were performed in a MicroAmp Optical 384-Well reaction plate (Applied Biosystems, Foster City, CA, USA) with Optical Adhesive Covers (Applied Biosystems, Foster City, CA, USA) in a Viia7 Real Time PCR System (Applied Biosystem, Foster City, CA, USA). Five serial dilutions of mixed DNAs were used to obtain the standard curve. The PCR volume for each sample was 10 μL, with 5 μL of SensiFASTTM SYBR^®^ Lo-ROX Kit (BIO_94020, Bioline), 1 μL of DNA template (4 ng dilution of each template) and 4 μL of 0.7 μM oligo mix (forward and reverse primers). The program reaction used was: 95 °C for 20s, 40 cycles of 95 °C for 1s and 60 °C for 20s. The program was set to reveal the melting curve of each amplicon from 60 °C to 95 °C and read every 0.5 °C. Single peaks for all genes confirmed gene-specific amplification and the absence of primer-dimers. All RT-qPCR reactions were carried out in triplicate to capture intra-assay variability. Each assay included three no-template negative controls for each primer pair. The quantification of the samples was conducted using the standard curve with the qPCR software QuantStudio Real-Time PCR (Applied Biosystems, Foster City, CA, USA). See Supplementary Materials for standard and melt curves in Figures S4, S5 and S9 and primer efficiency in Tables S6, S8 and S11.

## Figures and Tables

**Table 1:** Statistics of raw sequence data from which metagenome-assembled genomes (MAGs) were recovered through de novo assembly.

Parameter Type	Base Pairs
Total number of reads before trimming	20,505,104
Total number of reads after trimming	19,519,539
Average read length	239.5
Number of reads in contigs	17,286,414
Number of contigs >5 kb	416
N50 (bp)	146,992
Assembly size (Mb)	22.15
Maximum contig length (bp)	952,220

**Table 2:** Statistics for selected cluster genomes.

Cluster	Cl-1	Cl-2	Cl-8
Completeness %	97.66	99.57	99.05
Contamination %	0.38	1.3	1.64
Number of contigs	81	20	80
N50 (bp)	98,035	327,203	346,266
Assembly size (Mb)	4.78	3.74	8.57
Maximum contig length (bp)	239,842	952,220	622,837
Average coverage	633.5	66.6	31.6
Number of CDS	4680	4101	7071
G + C%	62	56	45
Number of tRNAs	37	40	48

**Table 3:** Selected MAG phylogeny.

Cluster	Cl-1	Cl-2	Cl-8
Phylum	Proteobacteria	Proteobacteria	Bacteroidota
Family	*Rhodobacteraceae*	*Parvibaculaceae*	*Cyclobacteriaceae*
Genus	*Aestuariivita*	*Mf105b01*	*Roseivirga_A*
Average Amino acid Identity (AAI)	77.56%	90.05%	81.41%

**Table 4:** Biosynthetic gene clusters identified by PRISM and antiSMASH.

	Cluster	Cl-1	Cl-2	Cl-8
PRISM	n° of Biosynthetic clusters	7	1	3
	Type of Biosynthetic clusters	PKS, NRPS, acyl homoserine lactone, ectoine	PKS	Lasso peptide, PKS, NRPS
antiSMASH	n° of Biosynthetic clusters	9	1	8
	Type of Biosynthetic clusters	NRPS, hserlactone, ectoine, terpenes, T1PKS, Bacteriocin	Bacteriocin	NRPS, T1PKS, T3PKS, bacteriocin, terpenes, beta lactone

**Table 5:** Oligo sequences.

Primer Name	Forward Sequence	Reverse Sequence	Tm (°C)	Amplicon Length (bp)
C8_c608	GCTCCAGTGTTTTAACCGG	CCATCTATTCTGCCGACC	62.1/60.7	251
C8_c450	TCGCCAATACTGATTATGCT	GTCGTAGTTCCTAAGGTCAC	59.7/55.3	169
C1_c182	CTGATCTGTTATATGATGCGGA	GACATGACAGTGATGCATTG	61.3/60.2	161
C2_c82	GTATCAATATCGGGCAGTGT	CGATATTCCAAATGTGAGCG	58.9/63.0	243
E9F/U1510R (16s)	GAGTTTGATCCTGGCTCAG	GGCTTACCTTGTTACGACTT	60/53.1	1500

## Data Availability

All raw sequence reads were deposited in the sequence read archive on the Genbank database under BioProject number PRJNA613991 and BioSample number SAMN14423147. The assembled, binned contigs for each cluster were submitted to the NCBI Genbank database under the following accession numbers: cluster1 (JAAWWD000000000), cluster2 (JAAWWE000000000) and cluster 8 (JAAWWF000000000). The accompanying MIMAG data are provided in Tables S1–S3.

## Supplementary Materials

The following are available online at https://www.mdpi.com/article/10.3390/ijms222413199/s1.
