# Supplementary material for: Three Novel Bacteria Associated with Two Centric Diatom Species from the Mediterranean Sea, Thalassiosira rotula and Skeletonema marinoi
Source: Int J Mol Sci. 2021 Dec 7;22(24):13199. doi: 10.3390/ijms222413199 (PMC8706122; doi:10.3390/ijms222413199)
Supplement: Supplementary file 1 [file ijms-22-13199-s001.zip › ijms-1484441-supplementary.pdf]

Three novel bacteria associated with two centric diatom species from the Mediterranean Sea, *Thalassiosira rotula* and *Skeletonema marinoi*

Federica Di Costanzo[1,2], Valeria Di Dato[1*], Leonardo Joaquim van Zyl[3*], Adele Cutignano[1,4], Francesco Esposito[1], Marla Trindade[3+], Giovanna Romano[1+]

Supplementary Figures

Figure S1. Analysis of MAG secondary metabolite pathways predicted using antiSMASH.
Figure S2. Confirmation of Cls presence in diatoms xenic cultures.
Figure S3. Uncropped image showing the detection of MAG following the introduction of *S. marinoi* FE7-FL bacteria into axenic *T. rotula* FE80 cultures.
Figure S4. qPCR Standard Curve and its Melt Curve Plot with specific primers for the evaluation of Cl-1 abundance in SA fraction of cultures grown in different salinities.
Figure S5. qPCR Standard Curve and its Melt Curve Plot with specific primers for the evaluation of MAG abundance in SA-FL culture fractions.
Figure S6. Uncropped image showing the detection of MAG growth in different types of media.
Figure S7. OD measurement and visual inspection of cultures grown in different types of media.
Figure S8. Uncropped image showing MAG detection in a medium F/2¼ with and without DNase treatment over nine days.
Figure S9. qPCR Standard Curve and associated melt curve plot with specific primers for the evaluation of MAG abundance in different media.

Supplementary Tables

Table S1. General genome features and MIxS of the Cluster1
Table S2. General genome features and MIxS of the Cluster2
Table S3. General genome features and MIxS of the Cluster8
Table S4. PRISM *T. rotula* Metagenome-Assembled Genomes (MAG) Cluster annotation.
Table S5. qPCR quantization of Cl-1 abundance in SA fraction of cultures grown in different salinities.
Table S6. qPCR Slope, R^2 and % of Efficiency with specific primers for the evaluation of Cl-1 abundance in cultures with different salinities.
Table S7. qPCR quantization of MAG abundance in SA and FL fractions.
Table S8. qPCR Slope, R^2 and % of Efficiency with specific primers for the evaluation of MAG abundance in SA-FL culture fractions samples.
Table S9. qPCR quantization of MAG abundance in FE7-FL grown in different media.
Table S10. qPCR quantization of MAG abundance in FE7-FL grown in F/2¼ medium supplemented with DNase-treated sonicate.
Table S11. qPCR Slope, R^2 and % of Efficiency with specific primers for the evaluation of MAG abundance in different media.
Table S12. antiSMASH detection of biosynthetic clusters in the whole metagenome.
Table S13. List of primers used for amplification of the selected gene clusters.

Supplementary information: Sequences of the clusters-specific genes.

Supplementary Figures

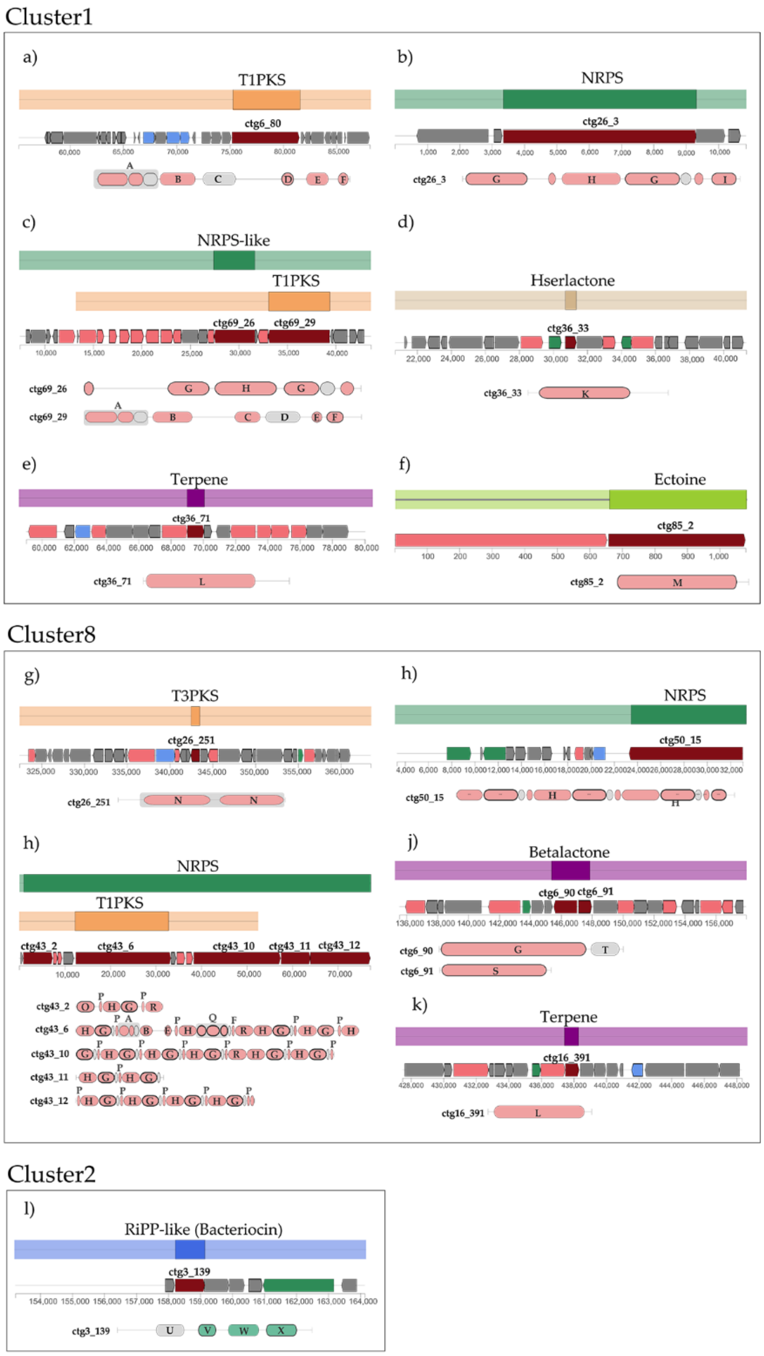

Figure S1. Analysis of MAG secondary metabolite pathways predicted using antiSMASH. Representative clusters organization of pathways related to Cluster1 *(a-f)*, Cluster8 *(g-k)* and Cluster2 (l). The genes constituting the clusters in the pathways are represented in different colors: red for core biosynthetic genes, pink for additional biosynthetic genes, blue for transport-related genes, green for regulatory genes and grey for other genes. The pfam domains constituting each core biosynthetic gene are also represented under the total cluster and are indicated by the letters ctg associated to numbers (i.e. ctg3_139). Domain abbreviations: A= ketoacyl synthase (KS), B= acyltransferase (AT), C= polyketide synthase dehydratase (PS-DH), D= Enoylreductase (ER), E= ketoreductase (KR), F= phosphopantetheine attachment site (PP), G= AMP-binding, H= condensation, I= thioesterase (TE), J= Bac_luciferase, K= Autoinducer synthase, L= squalene/phytoene synthase (SQS_PSY), M= Ectoine synthase, N= Chalchone and stylbene synthase, O= Co-enzyme A ligase (CAL), P= Peptidyl carrier protein (PCP), Q= Adenylation domain with integrated oxidase domain (A-OX), R= Epimerization, S=HMGL-like, T= C-terminal, U= Pkinase domain-containing protein 9 (PAS_9), V= His Kinase A (phosphoacceptor) domain (HisKA), W= Histidine kinase-like ATPases (HATPase c), X= Response regulator.

a

b

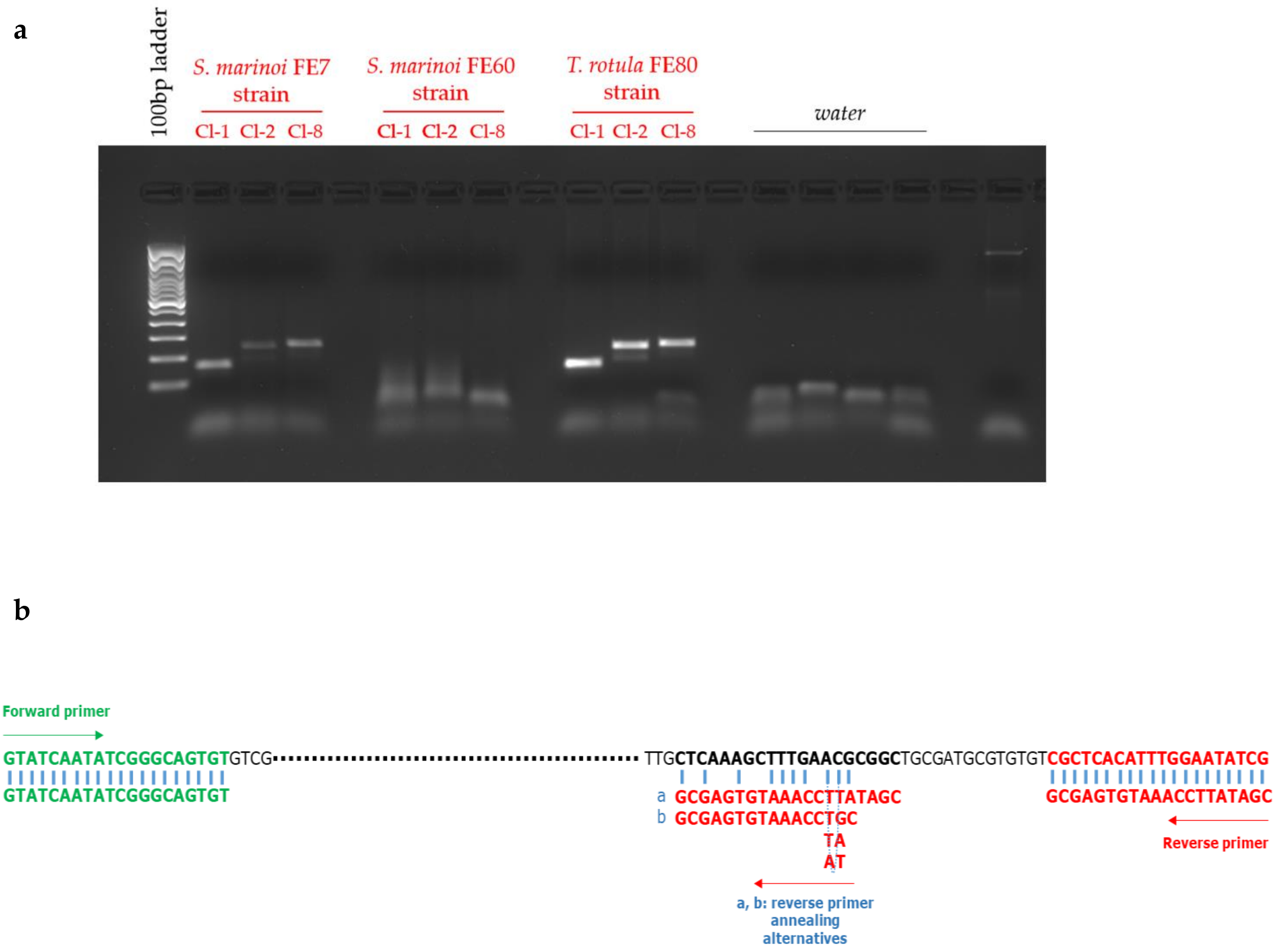

Figure S2. Confirmation of Cls presence in diatoms xenic cultures.

a. Uncropped image showing the MAG presence in different diatom species and strains. PCR amplification from 50ng of free-living bacterial fraction (FL) genomic DNA (gDNAs) with MAG-specific primers. gDNAs were extracted from the FL fraction of *Thalassiosira rotula* FE80 strain, *Skeletonema marinoi* FE7 and FE60 strains harvested at day 7 of growth. Abbreviations: Cl-1= Cluster1, Cl-2= Cluster2, Cl-8= Cluster8. 100bp ladder: from bottom to top: 100, 200, 300, 400, 500, 600, 700, 800, 900, 1000, 1200, 1500, 2000, 3000 bps.

b. Cl-2 reverse primer alternative annealing sites responsible for the lower band visible in panel **a-** lanes *S. marinoi* FE7 strain and *T. rotula* FE80 strain, Cl-2. The reverse primer has some aspecific annealing possibilities forward in the sequence, in respect to the original primer site chosen for the amplification. Sequencing of the lower band confirmed its specificity as Cl-2 contig_82 and its derivation from a second annealing site of the reverse primer.

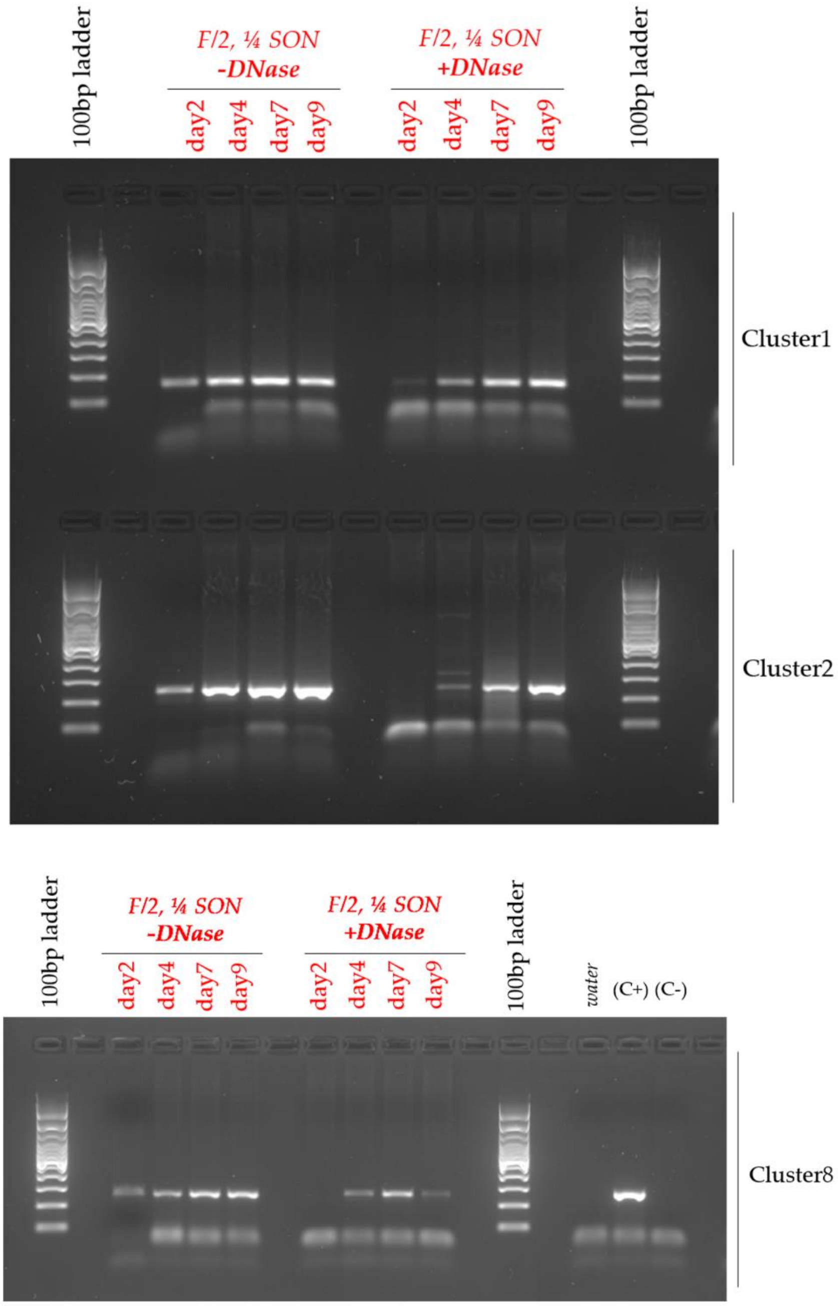

Figure S3. Uncropped image showing the detection of MAG following the introduction of *S. marinoi* FE7-FL bacteria into axenic *T. rotula* FE80 cultures. MAG PCR amplification from 50ng of strictly-associated (SA) and FL fractions gDNAs collected from *T. rotula* FE80 cultures in which FE7-FL bacteria were reintroduced. MAG presence was inspected after 1 (FE80_1), 3 (FE80_3), 6 (FE80_6) and 8 (FE80_8) months from the bacterial reintroduction. All the cultures were harvested at day 7 of growth. Abbreviations: (C-) = mixed gDNA from FE80 culture in which bacteria were not added, (C+) = mixed gDNA from FE7 culture. 100bp ladder: from bottom to top: 100, 200, 300, 400, 500, 600, 700, 800, 900, 1000, 1200, 1500, 2000, 3000 bps.

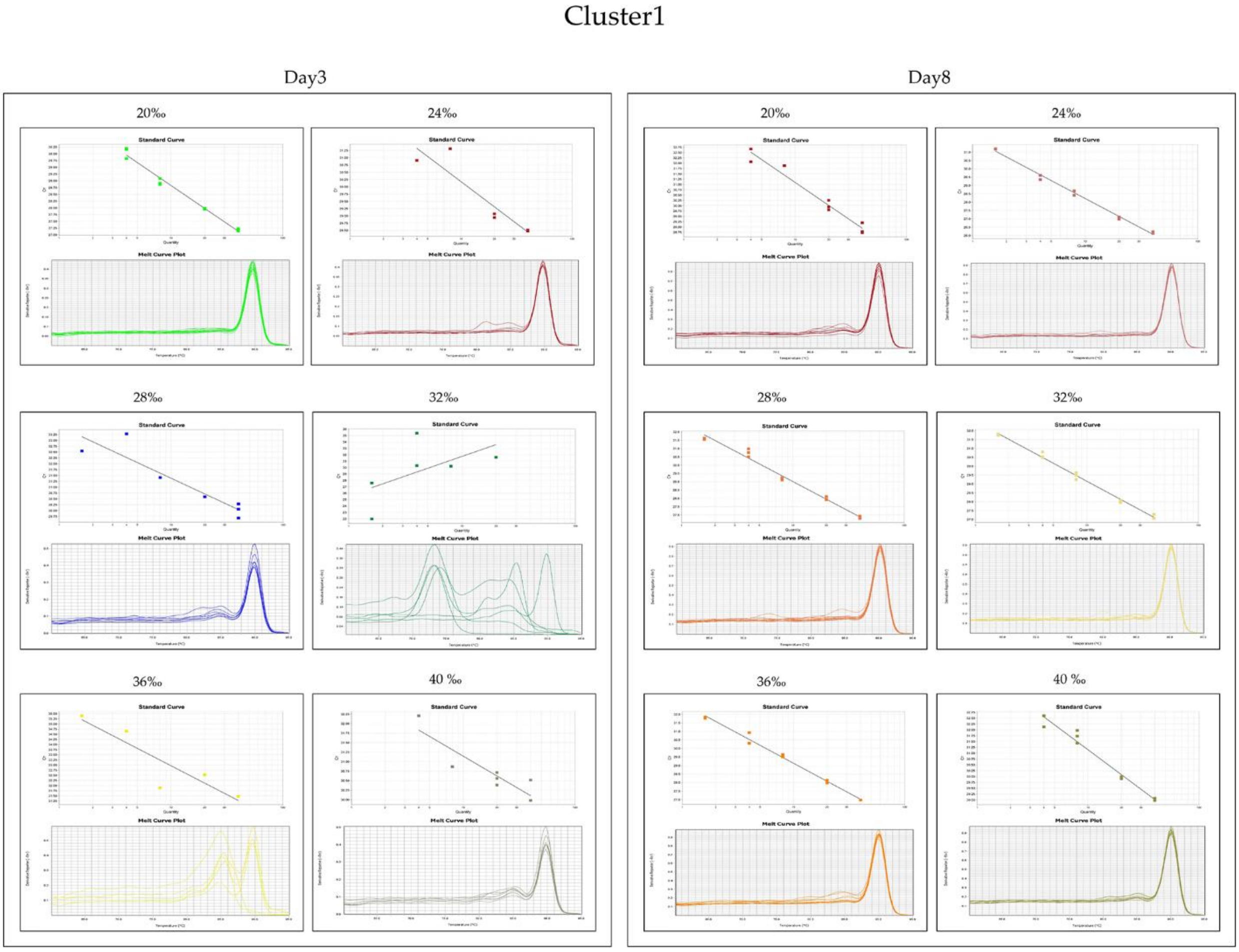

Figure S4. qPCR Standard Curves and their Melt Curve Plots with specific primers for the evaluation of Cl-1 abundance in SA fraction of cultures grown in different salinities. A standard curve was made for each salinity both at day 3 and day 8 of growth.

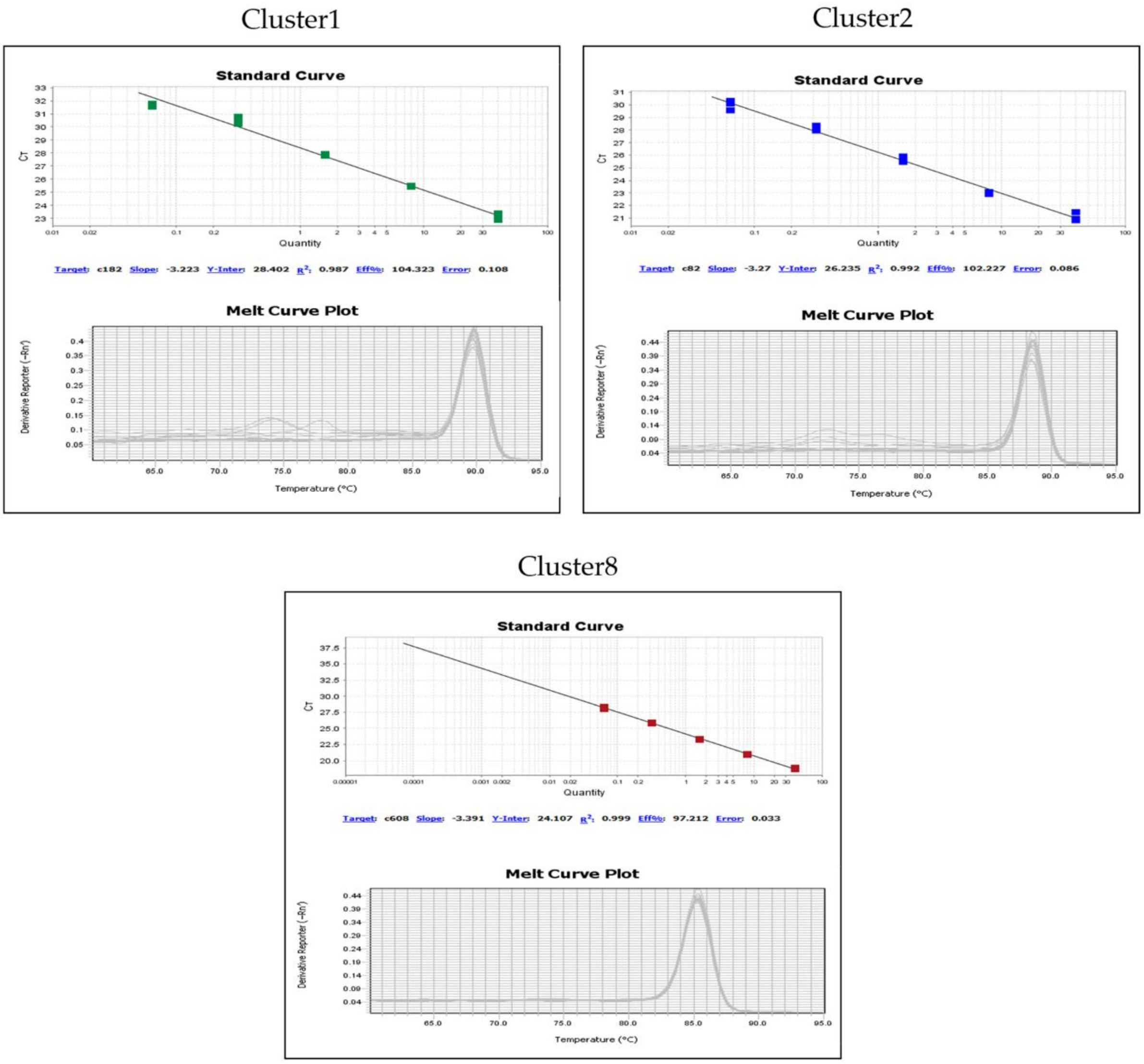

Figure S5. qPCR Standard Curve and its Melt Curve Plot with specific primers for the evaluation of MAG abundance in SA-FL culture fractions

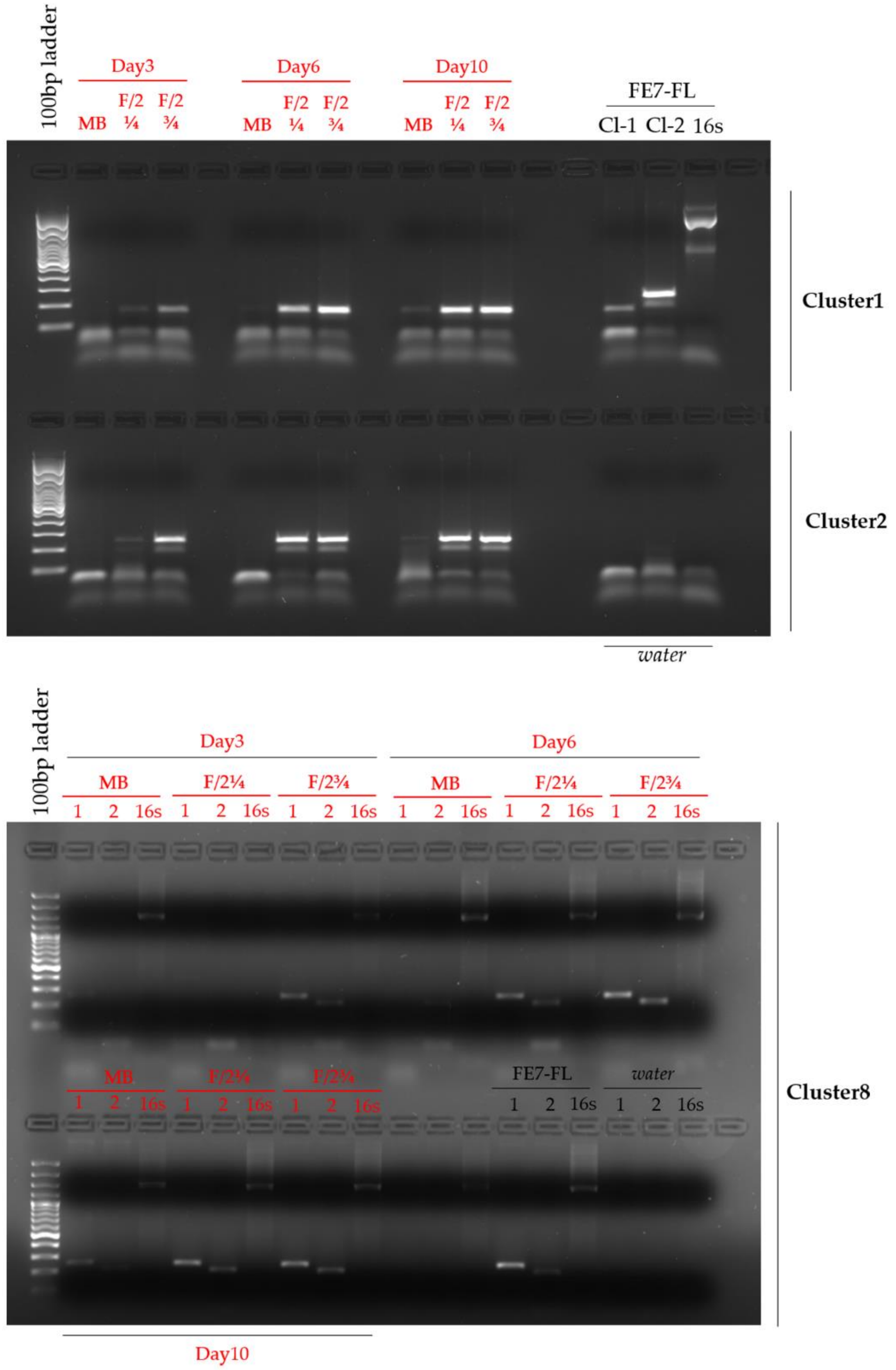

Figure S6. Uncropped image showing the detection of MAG growth in different types of media. PCR amplifications with MAG-specific primers on gDNA (50ng) of FE7-FL grown in the following types of medium: Marine Broth supplemented with FE7 spent culture medium (MB), F/2 supplemented with two different sonicated diatoms concentrations (¼ and ¾). Bacterial growth was followed along ten days. Abbreviations: Cl-1= Cluster1, Cl-2= Cluster2, 16s= E9/ U1510 universal primer. For Cluster8 were used two primer pairs (1= C8_c988; 2= C8_c450). 100bp ladder: from bottom to top: 100, 200, 300, 400, 500, 600, 700, 800, 900, 1000, 1200, 1500, 2000, 3000 bps.

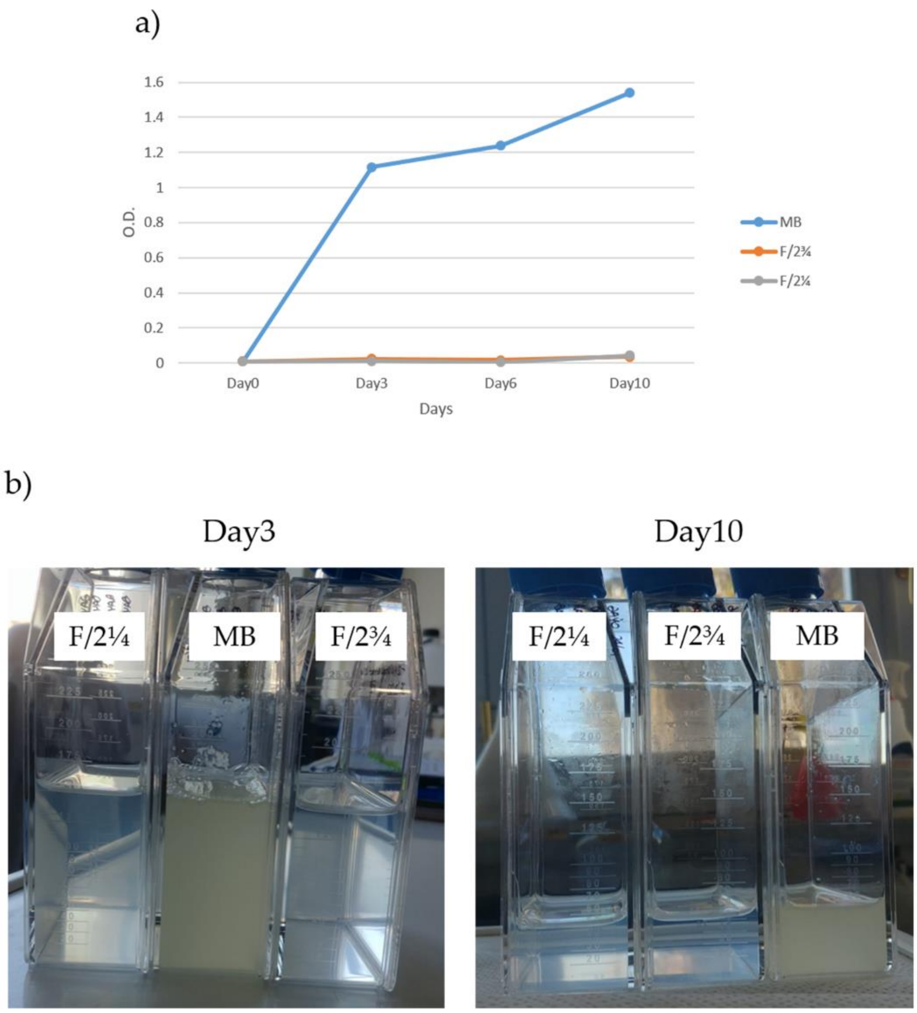

Figure S7. Optical density (OD) measurement and visual inspection of cultures grown in different types of media. **a)** OD reading of the FE7-FL total bacteria grown in MB or F/2 with the two sonicate concentrations. The OD were normalized on the blank (only MB or only F/2 without bacteria) and the means of three reads for each time point are reported in the graph; **b)** Visual inspection of the total FE7-FL bacteria growing in the three different media at day3 (starting sampling point) and day10 (final sampling point).

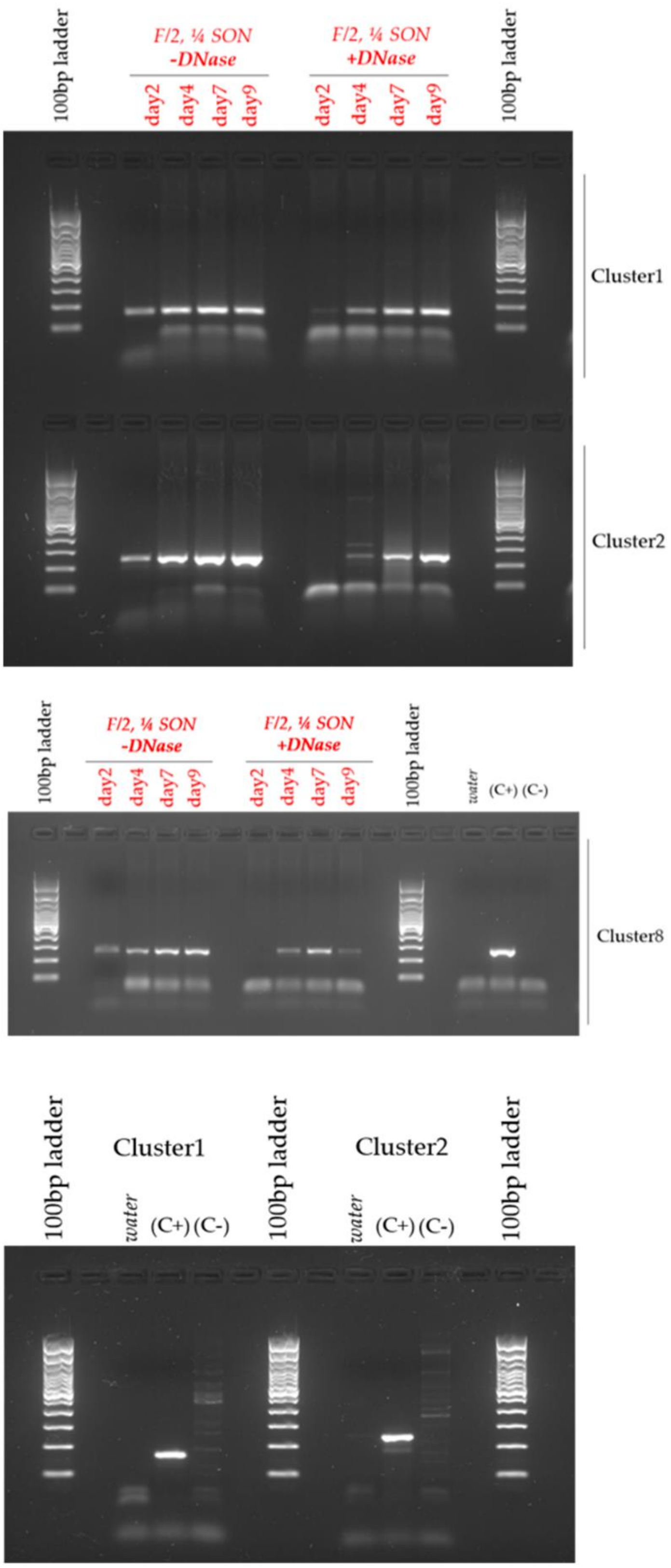

Figure S8. Uncropped image showing MAG detection in a medium F/2¼ with and without DNase treatment over nine days. PCR-based MAG detection in medium F/2¼ with and without DNase treatment over nine days. PCR amplifications with MAG-specific primers on gDNA (50ng) from FE7-FL bacteria grown in F/2¼ medium with (+DNase) and without (-DNase) the addiction of DNase. MAG presence was evaluated along nine days of culture growth. Abbreviations: (C-)= mixed gDNA from FE80 culture before the reintroduction of the bacteria, (C+)= mixed gDNA from FE7 culture. 100bp ladder: from bottom to top: 100, 200, 300, 400, 500, 600, 700, 800, 900, 1000, 1200, 1500, 2000, 3000 bps.

Cluster1

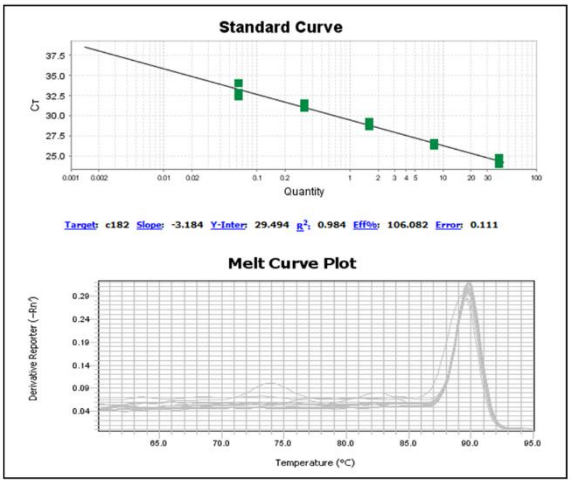

Cluster2

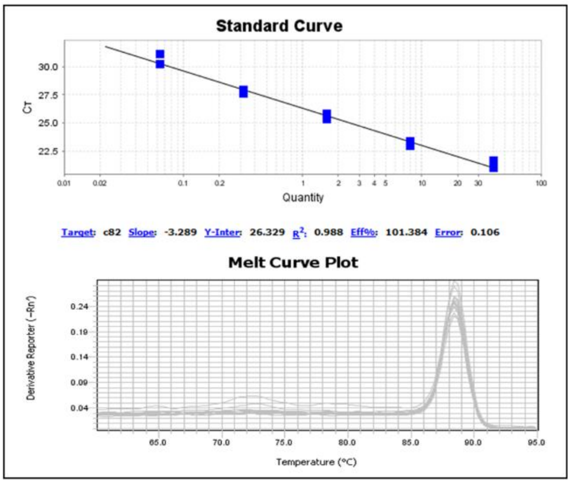

Cluster8

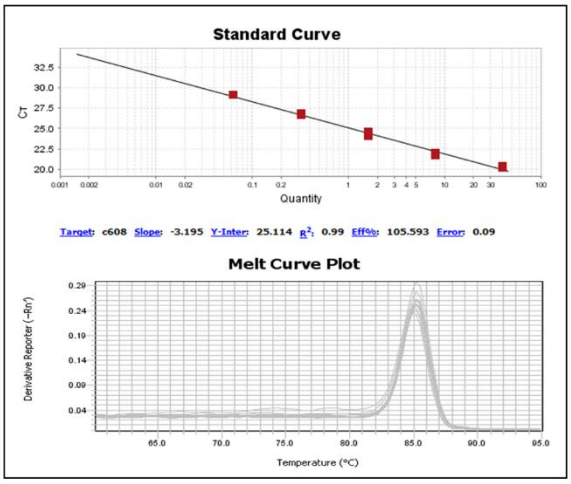

Figure S9. qPCR Standard Curve and its Melt Curve Plot with specific primers for the evaluation of MAG abundance in different media cultures.

Supplementary Tables

Table S1. General genome features and MIxS of the Cluster1.

Items	Description
**General features**	
**Classification Cluster1**	Domain *Bacteria*<br>Phylum *Proteobacteria*<br>Class *Alphaproteobacteria*<br>Order *Rhodobacterales*<br>Family *Rhodobacteraceae*<br>Genus *Aestuariivita*<br>Species *sp.*
**Gram stain**	Negative
**Cell shape**	NA
**Motility**	Motile
**Sporulation**	NA
**Temperature Range; Optimum**	18°C
**pH range; optimum**	NA
**Salinity range; optimum**	36ppm
**Carbon source**	NA
MIGS data	
**Investigation_type**	Bacteria
**Project_name**	Genome sequence of three bacteria associated with the diatom *Thalassiosira rotula*
**Lat_lon**	40°48.5′N, 14°15′E
**Depth**	NA
**Alt_elev**	NA
**Geo_loc_name**	Mediterranean sea: Central Tyrrhenian sea: East Sector
**Collection_date**	2011
**Env_biome**	marine biome [ENVO:00000447]
**Env_feature**	alga [ENVO:02500019]
**Env_material**	water [ENVO:00002006]
**Env_package**	host-associated environment [ENVO:01001000]
**Ref_biomaterial**	NA
**Pathogenicity**	NA
**Biotic_relationship**	Both free living and strictly associated
**Trophic_level**	Photoheterotroph
**Rel_to_oxygen**	Aerobic
**Isol_growth_condt**	NA
**Samp_store_temp**	18°C
**Sediment_type**	NA
Genome assembly data	

**Seq_meth**	Illumina MiSeq
**Assembly**	CLC Genomics
**Finishing_strategy**	N/A
**Annot_source**	PGAP (NCBI)

MAG Genome attribute

**Total number of reads before trimming**	20,505,104
**Total number of reads after trimming**	19,519,539
**Average read length**	239,5
**Number of reads in contigs**	17,286,414
**Number of contigs >5kb**	416
**N50 (bp)**	146,992
**Assembly size (Mb)**	22,15
**Maximum contig length (bp)**	952,220

Table S2. General genome features and MIxS of the Cluster2.

Items	Description
**General features**	
**Classification Cluster2**	Domain *Bacteria*<br>Phylum *Proteobacteria*<br>Class *Alphaproteobacteria*<br>Order *Parvibaculales*<br>Family *Parvibaculaceae*<br>Genus *Mf105b01*<br>Species *sp.*
**Gram stain**	Negative
**Cell shape**	NA
**Motility**	Motile
**Sporulation**	NA
**Temperature Range; Optimum**	18°C
**pH range; optimum**	NA
**Salinity range; optimum**	36ppm
**Carbon source**	NA
MIGS data	
**Investigation_type**	Bacteria
**Project_name**	Genome sequence of three bacteria associated with the diatom *Thalassiosira rotula*
**Lat_lon**	40°48.5′N, 14°15′E
**Depth**	NA
**Alt_elev**	NA
**Geo_loc_name**	Mediterranean sea: Central Tyrrhenian sea: East Sector
**Collection_date**	2011
**Env_biome**	marine biome [ENVO:00000447]
**Env_feature**	alga [ENVO:02500019]
**Env_material**	water [ENVO:00002006]
**Env_package**	host-associated environment [ENVO:01001000]
**Ref_biomaterial**	NA
**Pathogenicity**	NA
**Biotic_relationship**	Both free living and strictly associated
**Trophic_level**	
**Rel_to_oxygen**	Aerobic
**Isol_growth_condt**	NA
**Samp_store_temp**	18°C
**Sediment_type**	NA
Genome assembly data	
**Seq_meth**	Illumina MiSeq
**Assembly**	CLC Genomics

**Finishing_strategy**	N/A
**Annot_source**	PGAP (NCBI)

**MAG Genome attribute**	
**Total number of reads before trimming**	20,505,104
**Total number of reads after trimming**	19,519,539
**Average read length**	239,5
**Number of reads in contigs**	17,286,414
**Number of contigs >5kb**	416
**N50 (bp)**	146,992
**Assembly size (Mb)**	22,15
**Maximum contig length (bp)**	952,220

Table S3. General genome features and MIxS of the Cluster8.

Items	Description
**General features**	
**Classification Cluster8**	Domain *Bacteria*<br>Phylum *Bacteroidota*<br>Class *Bacteroidia*<br>Order *Cytophagales*<br>Family *Cyclobacteriaceae*<br>Genus *Roseivirga_A*<br>Species *sp.*
**Gram stain**	Negative
**Cell shape**	NA
**Motility**	Nonmotile
**Sporulation**	NA
**Temperature Range; Optimum**	18°C
**pH range; optimum**	NA
**Salinity range; optimum**	36ppm
**Carbon source**	NA
MIGS data	
**Investigation_type**	Bacteria
**Project_name**	Genome sequence of three bacteria associated with the diatom *Thalassiosira rotula*
**Lat_lon**	40°48.5′N, 14°15′E
**Depth**	NA
**Alt_elev**	NA
**Geo_loc_name**	Mediterranean sea: Central Tyrrhenian sea: East Sector
**Collection_date**	2011
**Env_biome**	marine biome [ENVO:00000447]
**Env_feature**	alga [ENVO:02500019]
**Env_material**	water [ENVO:00002006]
**Env_package**	host-associated environment [ENVO:01001000]
**Num_replicons**	
**Ref_biomaterial**	NA
**Pathogenicity**	NA
**Biotic_relationship**	Both free living and strictly associated
**Trophic_level**	Chemoorganotroph
**Rel_to_oxygen**	Aerobic
**Isol_growth_condt**	NA
**Samp_store_temp**	18°C
**Sediment_type**	NA
Genome assembly data	
**Seq_meth**	Illumina MiSeq

**Assembly**	CLC Genomics
**Finishing_strategy**	N/A
**Annot_source**	PGAP (NCBI)
**MAG Genome attribute**	
**Total number of reads before trimming**	20,505,104
**Total number of reads after trimming**	19,519,539
**Average read length**	239,5
**Number of reads in contigs**	17,286,414
**Number of contigs >5kb**	416
**N50 (bp)**	146,992
**Assembly size (Mb)**	22,15
**Maximum contig length (bp)**	952,220

Table S4. PRISM *T. rotula* Metagenome-Assembled Genomes (MAG) Cluster annotation.

				***T. rotula* MAG-Cluster1**			
**Biosynthetic Cluster**	PK		NRP	acyl homoserine lactone	Acyl homoserine lactone	PK, NRP	Ectoine
**Biosynthetic assemblies (ORF #)**	71, 75	56, 60, 62	37626, 3	33, 50261	25	23, 24, 26	2
**ORFs Domains**	71: Phosphopantetheinyltransferase; 75: Ketosynthase, Acyltransferase, Dehydratase, Enoylreductase, Ketoreductase, Thiolation.	56: Ketosynthase; 60: Thiolation; 62: Acyltransferase.	37626: Lasso peptide precursor; 3: Acyl adenylating enzyme, Thiolation, Condensation, Adenylation, Thiolation, Thioesterase.	33: Acyl homoserine lactone synthase; 50261: Dehydratase.	25: Acyl homoserine lactone synthase.	23: Phosphopantetheinyltransferase; 24: Formyltransferase, Adenylation, Adenylation, Thiolation; 26: Ketosynthase, Acyltransferase, Ketoreductase, Dehydratase, Thiolation, Thioesterase.	2: Ectoine synthase.
				***T. rotula* MAG-Cluster2**			
**Biosynthetic Cluster**	PK						
**Biosynthetic assemblies (ORFs #)**	352, 355, 356						
**ORFs Domains**	352: Acyltransferase; 355: Thiolation; 356: Ketosynthase.						
				***T. rotula* MAG-Cluster8**			
**Biosynthetic Cluster**	PK, NRP		NRP	lasso peptide			
**Biosynthetic assemblies (ORFs #)**	2, 3,_6,_8,_10,_11, 12		13, 17	178, 80751, 188, 80664, 191, 192			
**ORFs Domains**	2: Acyl adenylating enzyme, Thiolation, Condensation, Adenylation, Thiolation, Epimerization. 3: 2,3-diaminopropionate biosynthesis protein SbnA. 6: Condensation, Adenylation, Thiolation, Ketosynthase, Acyltransferase, Ketoreductase, Ketoreductase, Thiolation, Condensation, Adenylation, Adenylation, Thiolation, Epimerization, Condensation, Adenylation, Thiolation, Condensation, Adenylation, Thiolation, Condensation. 8: Amidotransferase. 10: Adenylation, Thiolation, Condensation, Adenylation, Thiolation, Condensation,		13: Thioesterase. 17: Condensation, Adenylation, Thiolation, Condensation, Adenylation, Thiolation, Condensation, Adenylation, Thioesterase.	178: Lasso peptide transglutaminase homolog; 80751: Putative lasso peptide precursor; 188: Sulfotransfera-se; 80664: Putative lasso peptide precursor; 191: Sulfotransfera-se; 192: Lasso peptide asparagine synthase homolog			

	Adenylation, Thiolation, Condensation, Adenylation, Thiolation, Epimerization, Condensation, Adenylation, Thiolation, Condensation, Adenylation, Thiolation.<br>11:<br>Condensation, Adenylation, Thiolation, Condensation, Adenylation.<br>12:<br>Thiolation, Condensation, Adenylation, Thiolation, Condensation, Adenylation, Thiolation, Condensation, Adenylation, Thiolation, Condensation, Adenylation, Thiolation, Condensation.

Table S5. qPCR quantization of Cl-1 abundance in SA fraction of cultures grown in different salinities.

Cluster1

Day3

	**20‰**		**24‰**		**28‰**	
**Sample name**	Quantity mean	Quantity SD	Quantity mean	Quantity SD	Quantity mean	Quantity SD
**Replicate_1**	14.5112409591674	0.798255980014801	8.16201210021972	0.606226086616516	N/D	N/D
**Replicate_2**	11.9483919143676	2.14905190467834	11.9473886489868	2.53087162971496	N/D	N/D
**Replicate_3**	12.2064056396484	3.06914186477661	12.0288705825805	3.67823576927185	N/D	N/D
	**32‰**		**36‰**		**40‰**	
**Sample name**	Quantity mean	Quantity SD	Quantity mean	Quantity SD	Quantity mean	Quantity SD
**Replicate_1**	N/D	N/D	N/D	N/D	N/D	N/D
**Replicate_2**	N/D	N/D	N/D	N/D	N/D	N/D
**Replicate_3**	N/D	N/D	N/D	N/D	N/D	N/D

Day8

	**20‰**		**24‰**		**28‰**	
**Sample name**	Quantity mean	Quantity SD	Quantity mean	Quantity SD	Quantity mean	Quantity SD
**Replicate_1**	31.6183595657348	5.80254477802653	6.68693971633911	0.831853449344635	11.7782106399536	3.32517790794372
**Replicate_2**	10.9883317947387	1.28338074684143	20.596580505371	1.56980264186859	39.6703758239746	1.72131192684173
**Replicate_3**	10.3919868469238	1.23993575572967	8.58546638488769	1.17350924015045	12.7557649612426	1.61978256702423
	**32‰**		**36‰**		**40‰**	
**Sample name**	Quantity mean	Quantity SD	Quantity mean	Quantity SD	Quantity mean	Quantity SD
**Replicate_1**	22.0469570159912	0.88727593421936	13.7929029464721	1.13168704509735	20.7034702301025	2.02587294578552
**Replicate_2**	14.0322103500366	3.17058825492858	30.1682376861572	3.14465661499737	11.9415206909179	0.597728669643402
**Replicate_3**	15.5249853134155	1.89186143875122	26.3027057647705	2.68924212455749	20.1522417068481	1.68568107645698

N/D – Not detected

Table S6. qPCR Slope, R^2 and % of Efficiency with specific primers for the evaluation of Cl-1 abundance in cultures with different salinities.

Cluster1

	**Day3**			**Day8**		
**Standard curves**	**Slope**	**R^2**	**Efficiency (%)**	**Slope**	**R^2**	**Efficiency (%)**
**20‰**	-2.816	0.983	126.497	-3.574	0.962	90.458
**24‰**	-2.877	0.872	122.655	-3.564	0.994	90.792
**28‰**	-2.228	0.852	181.147	-3.536	0.982	91.763
**32‰**	6.121	0.332	-31.351	-3.366	0.991	98.197
**36‰**	-2.812	0.803	126.801	-3.503	0.992	92.965
**40‰**	-1.722	0.799	280.820	-3.538	0.971	91.717

Table S7. qPCR quantization of MAG abundance in SA and FL fractions. Quantity Mean and Quantity Standard Deviations (SD) of the three MAG calculated via qPCR data analysis. Abbreviations: SA= strictly associated bacterial fraction; FL= free-living bacterial fraction; Day4= fourth day of culture growth; Day7= seventh day of culture growth.

Cluster1

	SA		FL	
**Days**	Quantity Mean	Quantity SD	Quantity Mean	Quantity SD
**Day4**	2.850962639	0.088266894	3.204114914	0.439069539
**Day7**	9.169445038	1.758309007	1.82853353	0.187807962

Cluster2

	SA		FL	
**Days**	Quantity Mean	Quantity SD	Quantity Mean	Quantity SD
**Day4**	4.156836987	0.520205617	0.114571519	0.026290052
**Day7**	12.14747334	0.741565466	0.185813785	0.033655368

Cluster8

	SA		FL	
**Days**	Quantity Mean	Quantity SD	Quantity Mean	Quantity SD
**Day4**	5.457804203	0.176898703	0.44285664	0.11048381
**Day7**	9.304740906	1.28759563	3.954962969	0.157003194

Table S8. qPCR Slope, R^2 and % of Efficiency with specific primers for the evaluation of MAG abundance in SA-FL culture fractions samples.

Primer name	Slope	R^2	Efficiency (%)
**Cluster8_c608**	-3.291	0.999	97.212
**Cluster2_c82**	-3.270	0.992	102.227
**Cluster1_c182**	-3.223	0.987	104.323

Table S9. qPCR quantization of MAG abundance in FE7-FL grown in different media.

Cluster1

	**MB**		**F/2¼**		**F/2¾**	
**Days**	Quantity Mean	Quantity SD	Quantity Mean	Quantity SD	Quantity Mean	Quantity SD
**Day3**	1.280346274	0.130211171	0.027051291	0.036026332	1.242784977	0.166221648
**Day6**	0.029285605	0.006642076	13.66777325	0.425333649	16.20821953	1.267161489
**Day10**	0.009822866	0.011881083	29.6822567	3.296260118	42.63631821	2.170769453

Cluster2

	**MB**		**F/2¼**		**F/2¾**	
**Days**	Quantity Mean	Quantity SD	Quantity Mean	Quantity SD	Quantity Mean	Quantity SD
**Day3**	0.032683714	0.016084398	0.024166935	0.002645122	4.377933025	0.241250709
**Day6**	0.061404854	0.039326753	18.35878563	0.782420158	30.27388763	2.298482418
**Day10**	0.090489812	0.004574142	24.60587311	0.367527306	39.92933273	3.180368423

Cluster8

	**MB**		**F/2¼**		**F/2¾**	
**Days**	Quantity Mean	Quantity SD	Quantity Mean	Quantity SD	Quantity Mean	Quantity SD
**Day3**	0.1362793	0.038577229	0.008	0.00915	2.689586878	0.452325106
**Day6**	0.129597649	0.034835324	18.79345703	3.886913193	14.20588303	1.957057476
**Day10**	0.095607392	0.018735146	27.99685478	2.574785471	39.20956039	2.05914402

Table S10. qPCR quantization of MAG abundance in FE7-FL grown in F/2¼ medium supplemented with DNase-treated sonicate.

F/2¼ (sonicate treated with DNase)

	**Cluster1**		**Cluster2**		**Cluster8**	
**Days**	Quantity Mean	Quantity SD	Quantity Mean	Quantity SD	Quantity Mean	Quantity SD
**Day2**	0.096502073	0.042068169	N/D	N/D	0.000123611	6.98944E-05
**Day4**	0.410697669	0.012779716	N/D	N/D	0.195641135	0.02719987
**Day7**	1.694224715	0.283904761	4.922961235	0.426027685	0.537181735	0.017694673
**Day9**	2.03311801	0.131902337	8.805378914	0.84562999	0.418001503	0.036180586

N/D – Not detected

Table S11. qPCR Slope, R^2 and % of Efficiency with specific primers for the evaluation of MAG abundance in different media.

Primer name	Slope	R^2	Efficiency (%)
**Cluster8_c608**	-3.195	0.990	105.593
**Cluster2_c82**	-3.289	0.988	101.384
**Cluster1_c182**	-3.184	0.984	106.082

Table S12. antiSMASH detection of biosynthetic clusters in the whole metagenome.

**Whole metagenome biosynthetic clusters**	
**n° of Biosynthetic clusters**	**Type of Biosynthetic clusters**
2	Ectoine
5	Terpenes
3	T1PKS
1	T3PKS
9	NRPS
3	Bacteriocin
1	RRE-containing
2	hserlactone
1	betalactone
1	Ranthipeptide
1	arylpolyene
1	Redox-cofactor

Table S13. List of primers used for amplification of the selected gene clusters.

Primer Name	Forward Sequence	Reverse Sequence	Tm (°C)	Amplicon length (bp)
**C8_c988**	AGCTTCAAACGTATCGATCA	CTCAAACAGGCAATTGGATG	59.9/63.1	206
**C8_c608**	GCTCCAGTGTTTTAACCGG	CCATCTATTCTGCCGACC	62.1/60.7	251
**C8_c450**	TCGCCAATACTGATTATGCT	GTCGTAGTTCCTAAGGTCAC	59.7/55.3	169
**C1_c182**	CTGATCTGTTATATGATGCGGA	GACATGACAGTGATGCATTG	61.3/60.2	161
**C2_c82**	GTATCAATATCGGGCAGTGT	CGATATTCCAAATGTGAGCG	58.9/63.0	243
**E9/ U1510 (16s)**	GAGTTTGATCCTGGCTCAG	GGCTTACCTTGTTACGACTT	60/53.1	1500

Supplementary information: Sequences of the cluster-specific genes.

C8_c988

>Diatom_S1_L001_R1_001_(paired)_unmapped_reads_[Diatom_S1_L001_R1_001]
(paired)_trimmed_(paired)_not_merged_contig_988 Average coverage: 35.51

AGCTTCAAACGTATCGATCAAAAGCCACTTGGTCATACCCTCATATTTACGATAGTGGTGGTAAGAGTAGGTTTTTCCAT
CTTCTGAAAAGACATGCTCCCTGCGTACCTCAAACTTTCCGGTCTCGTCCTTCACAGAGTCTTCATACAGGAGTTTTTTA
CCTTTGAATTTCAAAGAGATTTGATCCATCCAATTGCCTGTTTGAG

C8_c608

>Diatom_S1_L001_R1_001_(paired)_unmapped_reads_[Diatom_S1_L001_R1_001]
(paired)_trimmed_(paired)_not_merged_contig_608 Average coverage: 28.01

GCTCCAGTGTTTTAACCGGTTCTGGTTCAGGAGCTGGGGTGATTGCCTCTTTTGTTTCCTGTCCTGCTTCCGTTTTAGCC
TCTTCTTCTTTTTGCTCTTTTAGTGCCGCTGGTGCCGGCACTATAGGCGCTACTGCCACTGGCGTTTCAACCTGAGCAAC
GGCAACGACCGTCTCTTCTACAACGGTTGCAAAATCATGCTCATCCATAATAATGGCTTTGAGCAAGGCACGGTCGGCAG
AATAGATGG

C8_c450

>Diatom_S1_L001_R1_001_(paired)_unmapped_reads_[Diatom_S1_L001_R1_001]
(paired)_trimmed_(paired)_not_merged_contig_450 Average coverage: 39.94

TCGCCAATACTGATTATGCTTTAAGTAATGCTACCATTACCATTCTGGACGGAGCCACAACCGGTATGATCACTTTTACT
ATTCAGGATGATGCGGATATTGAAGGAGACGAGACCGCTACTTTGACGCTCAGCAATCCATCAGCAGGTGTGACCTTAGG
AACTACGAC

C1_c182

>Diatom_S1_L001_R1_001_(paired)_unmapped_reads_[Diatom_S1_L001_R1_001]
(paired)_trimmed_(paired)_not_merged_contig_182 Average coverage: 738.28

CTGATCTGTTATGATGCGGAATTCCCCTTGCTGGCGCGCGCGCTGGCGGAGGCCGACATCATTCTGGTGCCCTCGTGCAC
CGACGCGTTGTCGGGCTATTGGCGGGTGCGAATCGGGGCTATGGCCCGCGCGTTGGAAGGGCAATGCATCACTGTCATGT
C

C2_c82

>Diatom_S1_L001_R1_001_(paired)_unmapped_reads_[Diatom_S1_L001_R1_001]
(paired)_trimmed_(paired)_not_merged_ contig_82 Average coverage: 66.46

GTATCAATATCGGGCAGTGTGTCGAGAAGGGCGACGCGGGCGTCTTTCAGATTGGCGCGGGTATCGGCGAGCGCATGTTC
CGTTGACCAGCGCACATTGTCGAAGATCGAGCGCACATGAGGAAAACGCTTCAGGCCCACCAGCCAATAGCCGCCGTCTT
CCGCCGGCCCAAAGACAGCATCATGATTGCTCAAAGCTTTGAACGCGGCTGCGATGCGTGTGTCGCTCACATTTGGAATA
TCG
